# Cellular imaging by targeted assembly of hot-spot SERS and photoacoustic nanoprobes using split-fluorescent protein scaffolds

**DOI:** 10.1038/s41467-018-03046-w

**Published:** 2018-02-09

**Authors:** Tuğba Köker, Nathalie Tang, Chao Tian, Wei Zhang, Xueding Wang, Richard Martel, Fabien Pinaud

**Affiliations:** 1 Department of Biological Sciences, University of Southern California, 1050 Child Way, Los Angeles, CA 90089 USA; 2 Department of Chemistry, University of Montréal, C. P. 6128, Succursale Centre-Ville, Montréal, QC H3C 3J7 Canada; 3 Department of Biomedical Engineering, University of Michigan, 2200 Bonisteel Boulevard, Ann Arbor, MI 48109 USA; 4 Department of Chemistry, University of Southern California, 1050 Child Way, Los Angeles, CA 90089 USA; 5 Department of Physics and Astronomy, University of Southern California, 1050 Child Way, Los Angeles, CA 90089 USA

## Abstract

The in cellulo assembly of plasmonic nanomaterials into photo-responsive probes is of great interest for many bioimaging and nanophotonic applications but remains challenging with traditional nucleic acid scaffolds-based bottom-up methods. Here, we address this quandary using split-fluorescent protein (FP) fragments as molecular glue and switchable Raman reporters to assemble gold or silver plasmonic nanoparticles (NPs) into photonic clusters directly in live cells. When targeted to diffusing surface biomarkers in cancer cells, the NPs self-assemble into surface-enhanced Raman-scattering (SERS) nanoclusters having hot spots homogenously seeded by the reconstruction of full-length FPs. Within plasmonic hot spots, autocatalytic activation of the FP chromophore and near-field amplification of its Raman fingerprints enable selective and sensitive SERS imaging of targeted cells. This FP-driven assembly of metal colloids also yields enhanced photoacoustic signals, allowing the hybrid FP/NP nanoclusters to serve as contrast agents for multimodal SERS and photoacoustic microscopy with single-cell sensitivity.

## Abstract (web summary)

Traditional methods for the assembly of plasmonic nanoparticles into photo-responsive probes suffer from multiple problems. Here the authors use split fluorescent protein fragments as molecular glue to form stable nanoclusters for surface enhanced Raman scattering and photoacoustic imaging in live cells.

## Introduction

Noble metal gold (Au) and silver (Ag) nanoparticle (NPs) are particularly well suited to design optical probes for advanced biodetection and bioimaging applications because their nanoscale photophysical properties often surpass those of the best chromophores^1,2^. Their large optical cross-section, easy bio-functionalization and shape-tunable photo-response across the visible and near-infrared spectra have opened new imaging capabilities by surface plasmon resonance^3^, photoacoustic detections^4^ and surface-enhanced Raman scattering (SERS)^5^. When employed for SERS, plasmonic metal NPs provide highly sensitive optical detections of the vibrational signatures of Raman reporters positioned at or near their surface^6^. The strong near-field electromagnetic amplifications generated by optical excitation of metal NPs can indeed overcome the intrinsically low Raman cross-section of absorbed molecules and result in Raman scattering enhancement factors of 10^2^–10^12^ folds^7,8^ depending on the shape and the composition of NPs and on the number and the position of Raman reporters at their surface.

For targeted cell imaging by Raman scattering, SERS nanotags consisting of a spherical metal NP core pre-activated with thousands of surface Raman reporters are often used^9–11^. Such high-density coatings of the reporters and additional encapsulation in protective shells are required to compensate for the modest SERS enhancements of the NP core (10^2^–10^5^ folds) and to generate sufficient Raman signals for cell^12^ and in vivo imaging^13,14^. While anisotropic metal cores can improve Raman signals from nanotags^11^, SERS probes with superior detection sensitivity can be engineered by directed self-assembly of metal NPs into dimers or higher order nanoclusters and positioning of Raman reporters within interfacial nanogaps between NPs^15^. Upon clustering, interparticle plasmon-plasmon couplings at nanogaps between clustered NPs produce plasmonic hot spots where massive near-field amplifications in the range 10^8^–10^12^ folds enable single-molecule SERS detections^16–19^. Such high SERS enhancements are, however, strongly dependent on the stability of the Raman reporters within hot spots and on the size of the interparticle gap^15^, which requires significant optimization. Indeed, for nanogaps larger than 1–2 nm, near-field amplifications decay rapidly^20^ and for smaller nanogaps electron tunneling and field dissipation lower SERS enhancements^21^. Despite recent progress in NP assembly^22,23^, forming plasmonic hot spots reproducibly and precisely positioning biocompatible Raman reporters at these sites remains challenging and, compared to SERS nanotags^9^, bioimaging applications using SERS nanocluster probes having controlled hot-spot geometries remain limited despite their significant advantages for ultra-sensitive detections^18,24–26^.

In addition to providing versatile plasmonic platforms for SERS, metal NPs are also good exogenous contrast agents for photoacoustic detection of targeted cells and tissues^27,28^ where optical excitations induce transient thermal expansions around NPs and generate acoustic pressure waves detectable by ultrasound imaging^29,30^. In particular, AuNP clusters formed by DNA scaffold assembly^31^, biotin/avidin interactions^32^, or after cellular endocytosis^33^, have been shown to significantly enhance photoacoustic signals through increased rates of heat transfer and thermal coupling between AuNPs in close proximity compared to individual AuNPs. The clustering of metal NPs, especially if it is induced upon specific NP targeting to cells, as presented in this report, can thus provide enhanced photoacoustic imaging specificity in biological settings while simultaneously allowing SERS detection.

A promising approach for the controlled bottom-up assembly of metal nanoclusters having well-defined nanogaps and pre-programmed hot spots for SERS imaging and allowing enhanced photoacoustic detections is to employ Raman reporters that also act as molecular glue, for instance using host-guest interactions between complementary molecules appended to the surface of different NPs^34^. This strategy has been used to assemble NP SERS beacons, where nanoclustering driven by complementary nucleic acid scaffolds enhances the Raman scattering of chromophores pre-encoded at the surface of NPs or within the scaffold itself^35–38^. These approaches, however, suffer from multiple drawbacks, including (i) background SERS or fluorescence signals from the reporters, (ii) limited control of the nanogap size due to the lack of structural rigidity of nucleic acid scaffolds, and (iii) difficulties to carry out such assemblies in cells. Indeed, while nucleic acids remain the building blocks of choice for the in vitro assembly of colloidal NPs into photonic nanomaterials^39,40^, these scaffolds are degraded by nucleases and are very susceptible to pH and ion concentrations in biological buffers. Although peptide nucleic acid scaffolds can offset some of these issues^41^, specificity and binding affinity are anti-correlated in the one-dimensional zipping mechanism that underlies selective interactions between nucleic acids, which further limits their use for the remote assembly of SERS and photoacoustic NP clusters in cells and in vivo.

These limitations, however, can be overcome using other bio-inspired scaffolds such as self-complementary proteins and peptide fragments, whose secondary and tertiary structures provide unmatched binding specificity and affinity^42^. Among those, fluorescent proteins (FPs) offer many advantages to control the supramolecular assembly of metal NPs into photo-responsive nanoclusters. They are preprogramed to rapidly self-assemble from highly evolved protein domain building blocks and their folding mechanisms into compact and sturdy nanoscale entities is well understood^43^. They also encode biocompatible peptide-based chromophores that self-activate upon folding and whose peculiar Raman fingerprints can be differentiated from the vibrational signatures of other biomolecules^44,45^ with single-molecule SERS sensitivity^46^.

Recently, we assessed if complementary split fragments of the green fluorescent protein (GFP) could be employed as molecular glue to form metal NP clusters and theorized that the chromophore from GFP reconstructed within plasmonic hot spots might be used as a Raman reporter for SERS imaging^47^. Split GFP fragments are complementary domains from a super-folder GFP split into two highly asymmetric pieces^48^: a large GFP 1–10 domain (sGFP, amino acids 1–214) and a smaller M3 peptide domain corresponding the 11th β-sheet of the super-folder GFP β-barrel structure (M3, amino acids 215–230). Both fragments, including synthetic versions of the M3 peptide, spontaneously and irreversibly self-assemble in solution to form a fully folded GFP with a mature peptide-chromophore^49,50^. This bimolecular complementation system has been used to target nanomaterials in cells^49,51^ and to form protein nanostructures^52^. Here, we show that, when grafted on different metal colloids, FP fragments from GFP and its yellow (YFP) and cyan (CFP) spectral variants effectively guide the self-assembly of NPs into activatable SERS clusters and trigger the autocatalytic maturation of the FP chromophore within plasmonic hot spots that are homogenously seeded by a precise positioning of fully folded FPs at interfacial nanogaps. Upon dual-NP targeting to plasma membrane biomarkers in live cells, these NPs rapidly form discrete nanoclusters and the activation of the FP chromophore Raman signatures at plasmonic hot spots allows highly specific and site-directed SERS imaging of cancer cells. This in situ and FP-assisted clustering of metal NPs also yields strong photoacoustic signal amplifications, allowing the nanoclusters to serve as contrast agents for multimodal SERS and enhanced photoacoustic imaging of individual cells.

## Results

### Guided clustering of metal NPs using FP fragments

**Fig. 1:**
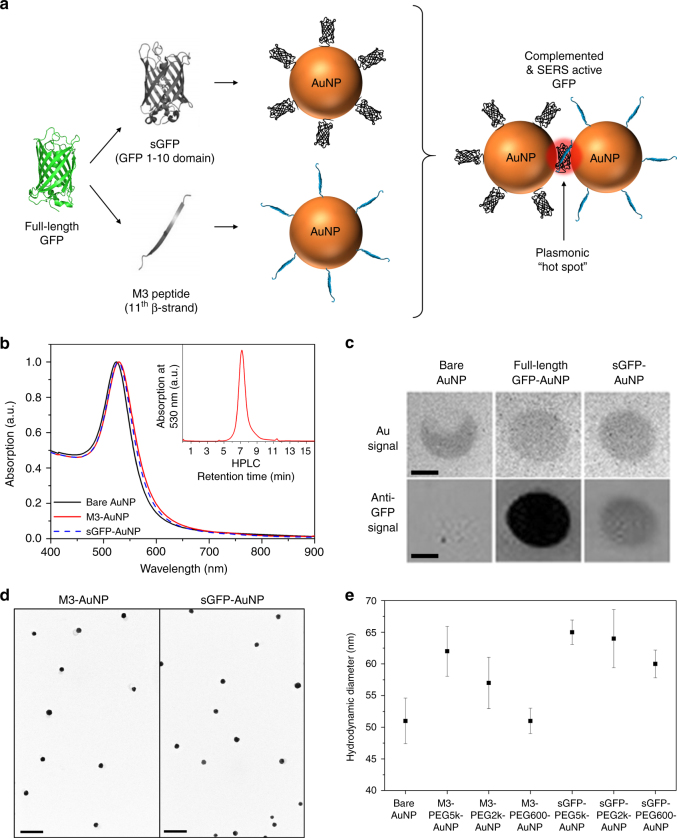
Characterization of AuNPs functionalized with split-fluorescent protein fragments. **a** Schematic of surface modification on AuNPs by sGFP and M3 peptide fragments and formation of SERS active hot spot through self-assembly and GFP complementation. **b** Comparison of the absorption spectra of bare AuNPs (solid black), AuNPs coated with M3 peptides (M3-AuNPs, solid red), and AuNPs coated with sGFP (sGFP-AuNPs, dash blue). Inset: size exclusion chromatography of M3-AuNPs. **c** Immuno-dot blot characterization of the presence of full-length GFP or sGFP at the surface of AuNPs. Scale bars: 0.5 cm **d** TEM images of monodispersed AuNPs coated with M3 peptides or sGFP. Scale bar: 200 nm. **e** DLS size distributions of bare AuNPs, M3-AuNPs and sGFP-AuNPs, coated with different size PEGs. The hydrodynamic diameter ( ± s.d.) was determined for *n* = 30 repetitive measurements for each sample

To exploit split FP fragments as surface molecular glue for the assembly of metal NPs (Fig. 1a) we first functionalized 40 nm AuNPs with either the large sGFP fragment or its complementary M3 peptide. sGFP-AuNPs were obtained by ligand exchange on citrate-stabilized NPs using a recombinant sGFP modified with a N-terminal tetracysteine motif^53^ and a flexible linker domain that allow its oriented binding and its conformational flexibility at the metal surface (Supplementary Fig. 1–3). A split-YFP fragment (sYFP) and a split-CFP fragment (sCFP) were also employed as substitutes of sGFP for coatings, and the full-length super-folder GFP (_fl_GFP) was used as a control. Complementary M3-AuNPs were functionalized with a synthetic M3 peptide fragment modified with a terminal cysteine for high affinity binding to the metal surface. For both sets of AuNPs, thiolated PEG-biotin (molecular weights of 5000, 2000, or 600 Da) was added during surface functionalization to increase the stability of the NPs. These coatings resulted in small spectral shifts of the localized surface plasmon resonance peak for both M3-AuNPs (*λ*_max_: 528 nm) and sGFP-AuNPs (*λ*_max_: 528 nm) compared to bare, citrate-stabilized AuNPs (*λ*_max_: 524 nm); a manifestation of local changes in refractive index typically observed after surface grafting of biomolecules^54^ (Fig. 1b). The stable surface anchoring of sGFP was confirmed by immuno-blotting assays with anti-GFP antibodies and clear immuno-reactivity was observed for sGFP-AuNPs compared to citrate-stabilized AuNPs (Fig. 1c). Interestingly, the absence of C-terminal 11th β-sheet in sGFP resulted in weaker immuno-reactivity for sGFP-AuNPs than _fl_GFP-AuNPs (Fig. 1c), suggesting that surface-bound sGFPs are oriented with their C-terminus exposed towards the buffer and accessible for binding complementary M3 peptides. The colloidal solutions of sGFP-AuNPs and M3-AuNPs were very stable with no signs of aggregation during size exclusion liquid chromatography (inset of Fig. 1b) and gel electrophoresis (Supplementary Fig. 4). In aqueous buffers, the zeta potential of sGFP-AuNPs was −14.1 ± 2.93 mV (mean ± s.d.) and that of M3-AuNPs was **−**22.8 ± 1.73 mV, an indication that, together with electrostatic repulsions, steric repulsions by M3 peptides, sGFPs and PEGs participate to the NP colloidal stability. The NPs were also monodispersed in transmission electron microscopy (TEM) images (Fig. 1d; Supplementary Fig. 5). Consistent with the expected influence that different FP fragments and different PEGs might have on the hydrodynamic diameter of functionalized NPs, the size of citrate-stabilized AuNPs (51 ± 4 nm) increased to 51–62 nm for M3-AuNPs and 60–65 nm for sGFP-AuNPs, depending on the length of PEGs employed during coating (Fig. 1e; Supplementary Fig. 6). Thus, various split FP fragments could readily be grafted and oriented at the surface of AuNPs with minimal impact on their final size, their photophysical properties and their colloidal stability.

**Fig. 2:**
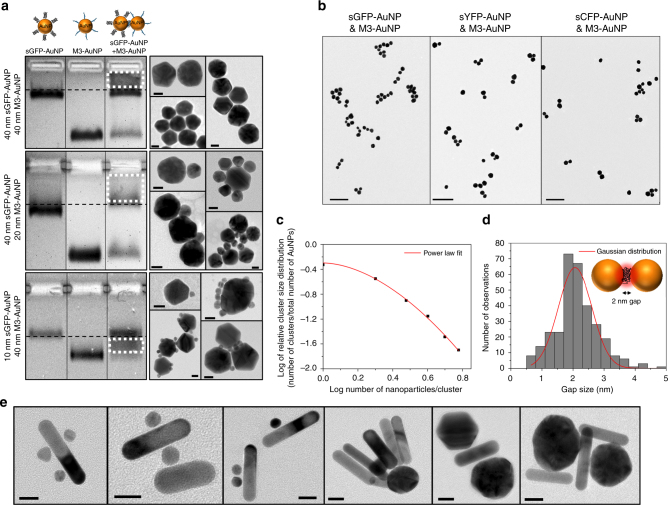
Formation of AuNP clusters by the assembly of FP fragments. **a** Agarose gel electrophoresis of sGFP-AuNPs, M3-AuNPs and unpurified sGFP-AuNPs + M3-AuNPs mixture and typical TEM images of clusters assembled with 40/40 nm AuNPs (top), 40/20 nm AuNPs (middle), and 40/10 nm AuNPs (bottom). White rectangles indicate the position of cluster bands in gels. Scale bars: 20 nm. **b** TEM images of clusters formed by the assembly of M3-AuNPs with sGFP-AuNPs, sYFP-AuNPs, or sCFP-AuNPs. Scale bars: 200 nm. **c** Normalized AuNP cluster size distribution and fit by a power law distribution model. **d** Size distribution of *n* = 314 nanogaps formed by the assembly of FP fragments between AuNPs in clusters. The distribution is Gaussian and centered at 2.1 ± 0.5 nm (mean ± s.d.). Inset: expected orientation of complemented GFP at nanogaps. **e** TEM images of clusters formed by the assembly of sGFP-gold nanorods with different sizes of M3-AuNPs. Scale bars: 20 nm

Taking advantage of the spontaneous self-assembly of M3 and sGFP fragments, we induced the clustering of AuNPs by simply co-incubating different sizes of M3-AuNPs and sGFP-AuNPs in buffer solutions. After 12 h, gel electrophoresis of the unpurified mixtures was performed and clusters were detected as smeared bands positioned at varying location in gels depending on the size of AuNP used in the reaction (40, 20, or 10 nm AuNPs, Fig. 2a, Supplementary Fig. 7). Typically, smears were accompanied by a lost, sometime only partial, of one or both M3-AuNP and sGFP-AuNP bands. When these smears were electro-eluted from gels, nanoclusters with various lengths and shapes were observed by TEM, including AuNP homodimers and heterodimers, AuNP chains, and more complexed two-dimensional and three-dimensional NP assemblies (Fig. 2a). The clustering process was solely dictated by the bimolecular complementation between the surface-attached FP fragments as confirmed by direct competition with an excess of free and non-cysteinilated M3 peptides (Supplementary Fig. 8). The complementation-driven assembly of clusters was also observed using the sYFP-AuNPs and sCFP-AuNPs spectral variants (Fig. 2b and Supplementary Fig. 9), an indication that the clustering process is broadly applicable to different sizes of NPs and to different types of split FP fragments.

Using 40 nm AuNPs, we then studied the kinetic of cluster formation. While some clusters were formed within an hour of co-incubation (Supplementary Fig. 10), the clustering kinetic was slow and at least 12 h co-incubation of M3-AuNPs with sGFP-AuNPs at room temperature was required to induce a clustering of more than 50% of all the NPs (Supplementary Fig. 11). Consistent with the competition gel assays, this slow kinetic suggested that binding and steric fit between complementary FP fragments, rather than diffusion-driven collisions of the NPs, is the primary mechanism of cluster formation. To characterize further this assembly process, we performed statistical analyses on the size heterogeneity of the AuNP clusters, which critically depends on whether the kinetic regime of clustering is diffusion-limited or reaction-limited^55,56^. After 12 h, 53% of all AuNPs formed clusters with at least two NPs. The assembly process was not random because the cluster size distribution could not be adequately described by a Poisson distribution model (F-test: *p* < 0.05, Supplementary Fig. 11). However, it followed a power-law probability distribution with an exponent of 1.7 ± 0.3 (Fig. 2c), as typically observed for irreversible and reaction-limited clustering of colloids in solution^55,56^. Thus, the slow formation of predominantly small and compact clusters observed by TEM (Fig. 2b; Supplementary Fig. 9) indicates that the clustering kinetic of M3-AuNPs with sGFP-AuNPs is primarily reaction limited, as expected for an assembly driven by the irreversible bimolecular complementation between M3 and sGFP fragments. This slow kinetic reflects the importance of steric fit between the complementary FP fragments, which have reduced conformational flexibility at the surface of AuNPs and whose molecular interactions are likely impacted by the three-dimensional degrees of freedom of the NPs diffusing in solution.

A salient characteristic of this split FP-driven assembly is that the interfacial gap size between clustered AuNPs should be uniform and controlled by the orientation of the re-assembled FP. To assess if this was indeed the case, we measured the dimension of more than 300 nanogaps between clustered AuNPs from TEM images. The resulting gap size histogram was well described by a Gaussian distribution centered at 2.1 ± 0.5 nm (Fig. 2d; Supplementary Fig. 12), a size that corresponds to the short axis of the GFP 2 nm × 4 nm cylindrical structure^57^ and is consistent with a transverse orientation of complemented GFPs at nanogaps (Fig. 2d). This observation, together with SERS measurements discussed below, indicates that the interparticle spacing within clusters is governed by the folding of the two split FP fragments into a re-assembled GFP at the interface between AuNPs. The formation of stable colloidal clusters was also efficient for a variety of other nanomaterials, including gold nanorods (Fig. 2e) and AgNPs (Supplementary Fig. 13). Thus, split FP fragments effectively act as molecular glue to guide the self-assembly of clusters having well-defined and small nanogaps for a variety of plasmonic nanomaterials.

### GFP chromophore activation within nanocluster hot spots

**Fig. 3:**
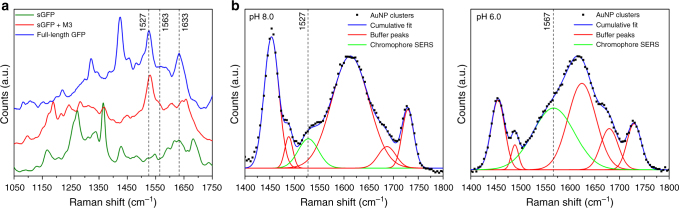
SERS spectra of complemented sGFP and assembled AuNP clusters. **a** SERS spectra of full-length GFP (blue), M3 peptide-complemented split-GFP (red), and non-complemented sGFP fragment (green) on 5 nm silver island substrates. **b** Liquid SERS spectra of assembled AuNP clusters at pH 8.0 (left) and pH 6.0 (right)

Beyond inducing clustering, the reconstruction of GFP between AuNPs is expected to trigger the formation of plasmonic hot spots and to activate the maturation of its chromophore within each nanogap. Indeed, once GFP is complemented, a tripeptide chromophore is rapidly formed by autocatalytic cyclization of three key amino acids residues within its β-barrel scaffold^43,48^. This cyclized and matured chromophore displays peculiar Raman fingerprints in the 1500–1650 cm^−1^ spectral region^44,45^. These include the C=C stretching mode of the exocyclic double bond at 1630 cm^−1^ and a normal vibrational mode delocalized over the imidazolinone ring and exocyclic C=C bond with bands at 1560 cm^−1^ (neutral) and 1530 cm^−1^ (anionic) depending on the ground-state protonation of the chromophore itself^44,45^. In Raman spectra of highly concentrated solutions of _fl_GFP, _fl_YFP, and _fl_CFP, these three main chromophore fingerprints were detected within ±10 cm^−1^ of their expected positions (Supplementary Fig. 14). For the sGFP fragment, which carries the three pre-cyclized amino acid residues, a detection of these vibrational signatures is not expected because the lack of 11th β-sheet precludes maturation. To establish that the Raman fingerprints of the chromophore are indeed absent in sGFP but can be activated upon complementation with M3 peptides, we used silver nanoisland plasmonic substrates and compared the SERS spectra of _fl_GFP with that of the sGFP fragment before and after complementation with M3 peptides. The peculiar vibrational modes of the mature GFP chromophore were detected at 1527 cm^−1^, 1563 cm^−1^, and 1633 cm^−1^ for the _fl_GFP and the M3-complemented sGFP but this triad was absent for the non-complemented sGFP alone (Fig. 3a) which confirmed the lack of chromophore cyclization in sGFP and established the possibility to activate its SERS signatures upon complementation with M3 peptides. Similar chromophore vibrational bands were detected when M3-AgNPs pre-incubated with sGFP fragments were added to the nanoisland substrates, an indication that chromophore activation is also efficient at the surface of metal colloids (Supplementary Fig. 15).

We then assessed if a similar SERS activation of the GFP chromophore fingerprints takes place within nanogaps in NP clusters assembled for 12 h using 40 nm M3-AuNPs and sGFP-AuNPs. Raman spectra of the _fl_GFP and SERS spectra of M3-AuNPs, sGFP-AuNPs or the assembled clusters were measured in buffers at pH 8.0 or 6.0 to promote the anionic or the neutral form of the chromophore, respectively. A non-resonance excitation at 785 nm was used to limit background fluorescence during Raman of _fl_GFP. For the _fl_GFP, which has a reported pKa of 5.5^48^, the Raman signature of the anionic chromophore imidazolinone/exocyclic C=C mode at pH 8.0 was observed at 1538 cm^−1^ and that of its neutral form at pH 6.0 was detected at 1549 cm^−1^, as previously reported for EGFP^58^ (Supplementary Fig. 16). For AuNP clusters, the SERS signature of the anionic chromophore was detected at 1527 cm^−1^ after deconvolution of vibrational modes from the surrounding pH 8.0 buffer (Fig. 3b). No GFP SERS signal was detected for colloidal solutions of sGFP-AuNPs or M3-AuNPs (Supplementary Fig. 16), indicating that the chromophore SERS fingerprint arises only when stable clusters are formed. SERS spectra of the AuNP clusters acquired at pH 6.0 displayed a shift of the 1527 cm^−1^ imidazolinone/exocyclic C=C mode toward 1567 cm^−1^ (Fig. 3b) as expected when the neutral form of the chromophore is favored over its anionic form at this lower pH^44,58^. Similar pH-dependent shifts of the GFP chromophore Raman signature were observed for AgNP nanoclusters (Supplementary Fig. 16), confirming that the re-assembly of split FP fragments into a mature GFP effectively results in SERS signal activation for different types of metal NP clusters. The cyclization of the GFP chromophore within assembled NP clusters also resulted in the appearance at 480 nm of the plasmonically enhanced optical absorption band of complemented GFP when differential absorption spectra of sGFP-AuNPs and M3-AuNPs mixtures at *t* = 0 min and *t* = 12 h were measured in an integrating sphere^59^ (Supplementary Fig. 17).

When accounting for the concentration of AuNPs, the cluster size distribution at 12 h and the respective Raman signal intensity of _fl_GFP and of complemented GFP in AuNP clusters, the experimental SERS enhancement factor of the chromophore 1527 cm^−1^ mode at pH 8.0 for a 785 nm excitation was 1.07 × 10^6^ fold. For comparison, the theoretical SERS enhancement factors of this vibrational mode calculated by finite-difference time-domain modeling for a similar size distribution of AuNP clusters with GFP-seeded plasmonic nanogaps of 2 nm or 4 nm were 2.39 × 10^6^ fold and 3.86 × 10^4^ fold, respectively; which correspond to a 2-fold and a nearly 30-fold difference with our measured experimental enhancement (Supplementary Fig. 18). When studied at 532 nm excitation, a similar 2-fold difference between experimental (1.32 × 10^6^ fold) and theoretical (2 nm gaps, 2.61 × 10^6^ fold) SERS enhancement factors of the GFP chromophore was observed. The good agreement between measured and theoretical SERS enhancements for the 2 nm nanogaps indicates that the GFP SERS fingerprint stems from the activation of its chromophore within uniform and 2 nm-sized plasmonic hot spots, as initially implied by our measured nanogap dimensions (Fig. 2d). Importantly, these data show that the split FP-guided assembly of AuNP clusters results in the formation of stable and well-defined hot spots homogenously seeded by a precise positioning of the complemented GFP within each nanogap. The absence of other protein or peptide Raman bands in the SERS spectra is additional evidence that controlled and uniform hot spots are formed within stable nanoclusters. Indeed, such modes were only detected when aggregation of the NPs was induced (Supplementary Fig. 19). The large experimental SERS enhancement factor obtained at non-resonant 785 nm or 532 nm excitations also demonstrates that the activated GFP chromophore within AuNP clusters can be detected with high sensitivity, as we previously predicted^47^. In these clusters, the concentration of complemented GFP was estimated at 0.6 nM, which implies that our SERS measurements in solution are performed in a single-molecule detection regime, where there is, in average, one GFP-seeded cluster in the detection volume at any time. For resonant excitation of the AuNPs clusters at 633 nm, the chromophore imidazolinone/exocyclic C=C mode at 1530 cm^−1^ is expected to be further amplified with SERS enhancement factors in the range of 10^8^–10^9^ fold^47^, a level of enhancement potentially sufficient for single-molecule SERS^16,60^. Thus, in addition to serving as molecular glue to assemble NP clusters with well-defined nanogaps, the complemented FP fragments also trigger the maturation of the GFP chromophore within uniform and small plasmonic hot spots. The activated chromophore effectively acts as a highly sensitive SERS reporter of the clustering process.

### Site-directed assembly of AuNP clusters in live cells

**Fig. 4:**
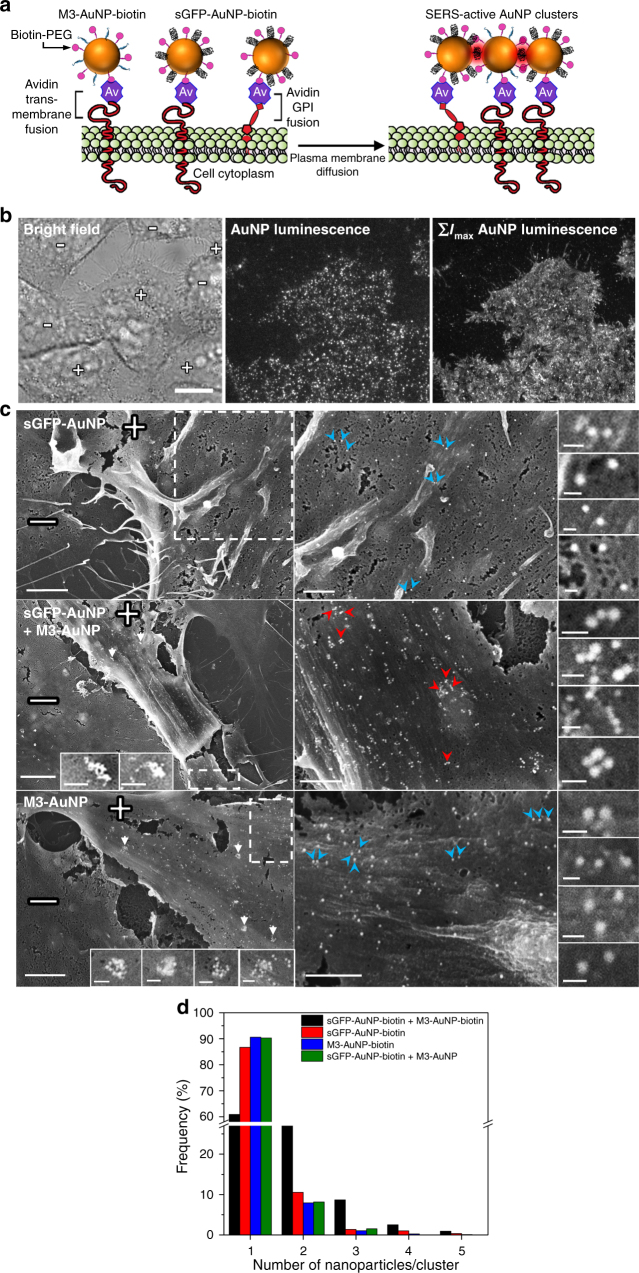
Cell targeting and plasma membrane clustering of biotinylated AuNPs. **a** Schematic of biotinylated M3-AuNPs and sGFP-AuNPs targeted to avidin fusion proteins at the plasma membrane of cells and their assembly into SERS active clusters. Not to scale. **b** Bright field (left), single-frame TIRF microscopy image (middle) and maximum intensity projection TIRF image from multiple frames (∑I_max_, right) of biotinylated M3-AuNPs bound to GPI-avidin fusion proteins and diffusing at the plasma membrane of live HeLa cells. Scale bar: 7 μm. **c** Scanning electron microscope images of U2OS cells co-expressing the transmembrane and the GPI-anchored avidin fusion proteins and targeted with biotinylated sGFP-AuNPs alone (top panel), biotinylated M3-AuNPs alone (bottom panel) or both biotinylated sGFP-AuNPs and M3-AuNPs simultaneously (middle panel). White arrows point towards endocytic membrane structures, blue arrowheads point towards AuNPs monomers and red arrowheads point towards some of the AuNP nanoclusters presented in insets. The plus and minus signs identify the avidin-expressing and non-expressing cells, respectively. Scale bars: 2 μm (left panels), 200 nm (insets of left panels), 1 μm (right panels), and 100 nm (insets of right panels). **d** Cluster size distributions of AuNPs on targeted U2OS cells

To test whether AuNP clusters could be assembled directly in live cells, we first used HeLa and U2OS cells expressing extracellular transmembrane and GPI-anchored avidin fusions^61^ and targeted these surface biomarkers with 40 nm M3-AuNPs and sGFP-AuNPs via their surface-attached biotin-PEG_600_ moieties (Fig. 4a). Both sets of biotinylated NPs rapidly recognized the avidin fusions on expressing cells (Supplementary Fig. 20) and the membrane-bound AuNPs could be imaged by total internal reflection fluorescence (TIRF) microscopy by exploiting non-linear luminescence from spherical AuNPs near borosilicate glass coverslips^62^. In the plane of the plasma membrane, individual biotin-M3-AuNPs or biotin-sGFP-AuNPs diffused rapidly and over the entire surface of expressing cells (Fig. 4b; Supplementary Movie 1), consistent with the expected rapid lateral mobility of the avidin fusions^49,61^. This indicated that after specific binding to plasma membrane biomarkers, AuNPs remain monodispersed and highly mobile, which is critical for their self-assembly into SERS nanoclusters at the cell surface.

When biotinylated M3-AuNPs and sGFP-AuNPs were simultaneous targeted on cells co-expressing both avidin fusions, a variety of linear clusters, including dimers, trimers, tetramers, and longer AuNPs chains, quickly formed at the cell surface and 40% of all the membrane-bound AuNPs were clustered within 1 h of incubation (Fig. 4c, d; Supplementary Table 1). More than 80% of the clusters formed at the plasma membrane were linear chains (Supplementary Fig. 21). In comparison, biotin-M3-AuNPs or biotin-sGFP-AuNPs targeted separately showed minimal clustering on cells, with 91 and 87% of the NPs remaining as monomers, respectively (Fig. 4c, d; Supplementary Table 1). We attributed the additional presence of a few large and circular AuNP aggregates to early stage endocytic events, because they were also detected on cells incubated with only biotin-M3-AuNPs (insets of Fig. 4c). For cells targeted with either M3-AuNPs or sGFP-AuNPs lacking biotin-PEG_600_ moieties, 90 % of the membrane-bound NPs remained monomeric (Fig. 4d; Supplementary Fig. 22 and Supplementary Table 1), an indication that the rapid clustering of targeted AuNPs is dependent on their binding and lateral diffusion at the cell plasma membrane and that the clusters are not produced by a pre-assembly of AuNPs in the cell media. Replacing biotin-PEG_600_ by biotin-PEG_2000_ on AuNPs did not influence the clustering efficiency on cells (Supplementary Fig. 21 and 23). However, when biotinylated AuNPs were co-targeted to cells expressing only the GPI-avidin fusion, clusters were mainly dimeric; suggesting that the type of membrane anchorage for the targeted biomarkers can influence the clustering process (Supplementary Fig. 24). At the plasma membrane, the assembly of targeted AuNPs is therefore driven by the bimolecular complementation of the split FP fragments and is not the result of random aggregations or accumulation in membrane pits or cavities. The kinetic of clustering on cells is much faster than in solution and is primarily assisted by the lateral diffusion of the targeted biomarkers. This is consistent with the fact that, for binding reactions that also depend on diffusion, the reaction efficiency is increased when the number of dimensions in which diffusion occurs is reduced. Indeed, the fast mobility of AuNPs and their diffusion within the two-dimensional plane of the cell membrane facilitate molecular interactions and steric fit between the surface-appended FP fragments and improve the clustering efficiency compared to assemblies in solution where AuNPs undergo three-dimensional and rotational diffusions. The self-assembly of AuNP clusters at the plasma membrane of cells did not induce any apparent cellular toxicity and avidin-expressing cells targeted by biotin-M3-AuNPs, biotin-sGFP-AuNPs or the assembled clusters showed good viability over a 48 h period in MTT cytotoxicity assays **(**Supplementary Fig. 25**)**.

### SERS microscopy imaging of nanoclusters assembled in cells

**Fig. 5:**
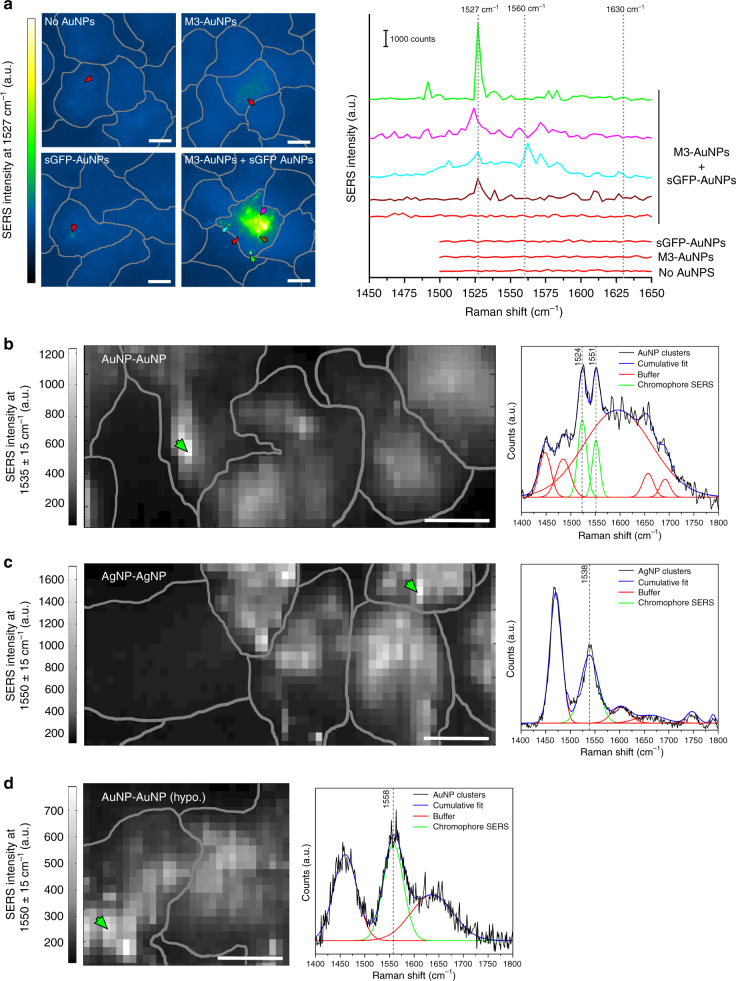
Targeted SERS imaging of cells with split-FP assembled metal nanoclusters. **a** SERS microscopy images of fixed cells at the GFP chromophore 1527 cm^−1^ imidazolinone/exocyclic C=C Raman mode and corresponding SERS spectra on cells expressing the avidin biomarkers and targeted by biotinylated M3-AuNPs and sGFP-AuNPs separately or simultaneously. Colored arrows in images point toward single pixels whose SERS spectra are represented in matching colors. The three typical GFP chromophore vibrational modes are indicated by dash lines on spectra. **b** SERS microscopy image of live cells co-targeted by biotinylated M3-AuNPs and sGFP-AuNPs and reconstructed at a 1535 ± 15 cm^−1^ spectral window. The deconvolved SERS spectrum corresponds to one pixel in the cell image as indicated by the arrow. **c** SERS image of live cells co-targeted by biotinylated M3-AgNPs and sGFP-AgNPs and reconstructed at a 1550 ± 15 cm^−1^ spectral window. The SERS spectrum corresponds to the individual pixel indicated by the arrow in the cell image. **d** SERS image of live cells in hypotonic buffer after co-targeting of biotinylated M3-AuNPs and sGFP-AuNPs and reconstruction at a 1550 ± 15 cm^−1^. The SERS spectrum corresponds to one pixel in the cell image as indicated by the arrow. All scale bars: 10 µm

Cells grown at confluency and transfected with both avidin biomarkers were then co-targeted for 1 h with 40 nm biotin-M3-AuNPs and biotin-sGFP-AuNPs and imaged live or after chemical fixation by SERS microscopy. Wide-field SERS imaging of fixed cells was performed in PBS buffer at pH 8.0 using a 532 nm excitation and a Raman hyperspectral imager based on Bragg tunable filters^63^. In the absence of AuNPs, no GFP chromophore fingerprints were detected in SERS spectra and only a diffuse background autofluorescence was observed when spectral images were reconstructed at 1527 cm^−1^ to localize the imidazolinone/exocyclic C=C Raman mode on cells (Fig. 5a). Likewise, no SERS fingerprints of the GFP chromophore were detected for cells targeted with only biotin-M3-AuNPs or only biotin-sGFP-AuNPs and spectral images at 1527 cm^−1^ displayed the cell background and a few low intensity spots attributed to weak photoluminescence signal contributions from individual AuNPs^64^ (Fig. 5a). In contrast, the typical vibrational signatures of the GFP chromophore were clearly visible in SERS spectra taken at various positions along the plasma membrane of cells co-targeted by both biotin-M3-AuNPs and biotin-sGFP-AuNPs (Fig. 5a), a direct confirmation that the biomarker-assisted clustering of AuNPs effectively induces the activation of the GFP chromophore within plasmonic hot spots at the cell surface. In these spectra, the three chromophore fingerprints were detected within ±10 cm^−1^ of their expected position, with the 1527 cm^−1^ imidazolinone/exocyclic C=C mode often dominating the signal (Fig. 5a). When images were reconstructed at this wavenumber, targeted cells were specifically distinguished from non-transfected cells that did not express the biomarkers (Fig. 5a; Supplementary Fig. 26). Although the SERS signal at 1527 cm^−1^ was detected across the cell surface, its intensity was not uniform. Large areas of the plasma membrane and smaller punctuates displayed high SERS signals while other membrane domains lacked the spectral fingerprints of GFP and show background signals similar to those observed in cells targeted with only biotin-M3-AuNPs or biotin-sGFP-AuNPs (Fig. 5a). This variation in SERS signal across targeted cells is consistent with the presence of residual non-clustered AuNPs at the plasma membrane (Fig. 4c) and with the size distribution of the clusters which can display different GFP SERS intensities^47^ (Supplementary Fig. 18**)**.

For live cell imaging, cells were incubated with 40 nm biotin-M3-AuNPs and biotin-sGFP-AuNPs for 1 h, washed and further imaged over a one-hour period in pH 8.0 PBS buffer by confocal Raman microscopy at 532 nm excitation. As for fixed cells, the imidazolinone/exocyclic C=C modes of the GFP chromophore were detected within ±10 cm^−1^ of their expected positions, at about 1524 cm^−1^ and 1551 cm^−1^ in SERS spectra of targeted cells (Fig. 5b). In images reconstructed at 1535 ± 15 cm^−1^ to encompass the Raman signals from both fingerprints, the SERS signal was diffused and SERS spectra from individual pixels were more noisy than in fixed cells (Fig. 5b), consistent with the expected mobility of the assembled AuNP clusters in live cells. Similar clustering-dependent activation of the GFP chromophore Raman fingerprints were obtained when 40 nm biotinylated M3-AgNPs and sGFP-AgNPs were co-targeted to cells and imaged under the same conditions (Fig. 5c). This confirmed that different types of metal NPs could self-assemble into hot spot SERS nanoprobes directly in live cells via in situ complementation of split FP fragments. With AgNP clusters, SERS signal-to-noise detections of the GFP chromophore fingerprints were significantly improved compare to AuNPs (Fig. 5c), because AgNPs have superior scattering efficiency than AuNPs and because plasmon-plasmon coupling between AgNPs red-shifts their maximum near-field enhancement wavelength^20,65^ in resonance with the 532 nm excitation used for cell imaging. To confirm that the detected 1530–1560 cm^−1^ SERS vibrational bands arise from the complementation of GFP, we also stimulated the early endocytosis of 40 nm biotin-M3-AuNPs and biotin-sGFP-AuNPs clusters by imaging targeted cells in an hypotonic PBS buffer^66^. Under this condition, the neutral GFP chromophore Raman signature at about 1560 cm^−1^ was primarily detected in SERS spectra, consistent with an accumulation of the nanoclusters into early endosomal compartments which have a slightly acidic pH of 6.0–6.5^67^ (Fig. 5d). Complementary metal NPs functionalized with split FP fragments can therefore rapidly self-assemble into active hot spot SERS nanocluster probes when co-targeted to diffusing plasma membrane biomarkers in live cells and the in situ activation of the GFP chromophore Raman fingerprints allows highly specific and single-cell SERS imaging.

### SERS imaging of cancer cells with FP-assembled AuNP clusters

**Fig. 6:**
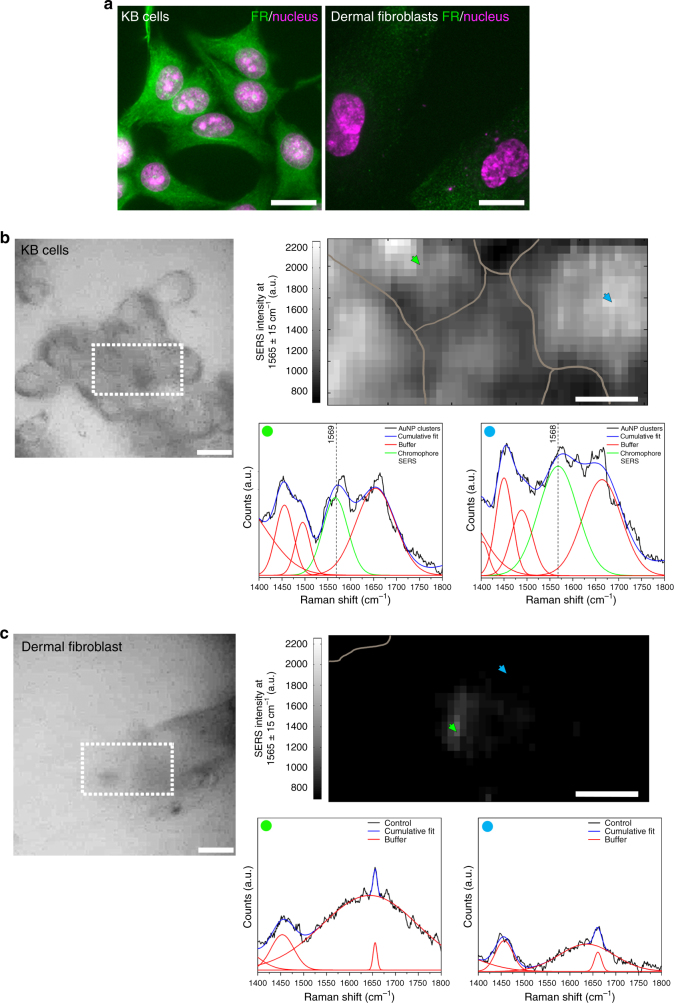
SERS imaging of cancer cells with folate-functionalized AuNPs. **a** Wide-field fluorescence imaging of folate receptor expression in human carcinoma KB cells (left) and human primary dermal fibroblasts (right). Scale bars: 20 µm. **b** Reflection image, SERS microscopy image reconstructed at a 1565 ± 15 cm^−1^ spectral window and SERS spectrum of individual pixels (colored arrows) for live carcinoma KB cells co-targeted by folate-functionalized M3-AuNPs and sGFP-AuNPs. Scale bars: 20 µm (left) and 10 µm (right). **c** Reflection image, SERS microscopy image reconstructed at a 1565 ± 15 cm^−1^ spectral window and SERS spectrum of individual pixels (colored arrows) for a live primary dermal fibroblast co-targeted by folate-functionalized M3-AuNPs and sGFP-AuNPs. Scale bars: 20 µm (left) and 10 µm (right)

To demonstrate that cancer cells could also be targeted and imaged by site-directed assembly of AuNP clusters, we targeted M3-AuNPs and sGFP-AuNPs to endogenous folate receptors (FR), which are cancer biomarkers overexpressed at the plasma membrane of many human cancers^68^. The functionalization of AuNPs with folate, a high affinity ligand for FR, was done by replacing biotin-PEG with folate-PEG. The assembly of 40 nm folate-M3-AuNPs and folate-sGFP-AuNPs was then tested on live human carcinoma KB cells, which overexpress FR, and on human primary dermal fibroblasts, which display normal FR expression^69^ (Fig. 6a). In SERS microscopy images acquired at 532 nm excitation, FR-overexpressing KB cells displayed the neutral GFP chromophore imidazolinone/exocyclic C=C mode at about 1560–1570 cm^−1^, consistent with an effective assembly of AuNP clusters after targeting and with their endocytosis inside cells, as typically observed for NPs targeted to surface FR^70^ (Fig. 6b). In comparison, human primary dermal fibroblasts labeled under the same conditions only showed cell background signals without the typical SERS signature of the GFP chromophore (Fig. 6c), indicating an absence of AuNP cluster formation due to the much lower FR expression in these cells compared to KB cells. Thus, consistent with the SERS imaging of cells expressing avidin fusions, split-FP-guided clustering of AuNPs targeted to endogenous and overexpressed biomarkers enables the specific detection of pathogenic cancer cells over normal primary cells by SERS imaging.

### Photoacoustic imaging of AuNP clusters on targeted cells

We then assessed if the close proximity between AuNPs in split-FP-assembled clusters could effectively provide enhanced photoacoustic detections compared to individual AuNPs after targeting to cells. For this, we used a custom laser scanning photoacoustic microscope^27^ equipped with a 532 nm nanosecond laser as an excitation source and with an ultrasonic transducer to capture excited photoacoustic waves from the AuNPs in cells. Avidin-expressing U2OS targeted with 40 nm biotinylated AuNPs or carcinoma KB cells and primary dermal fibroblasts targeted with 40 nm folate-functionalized AuNPs were imaged after chemical fixation.

**Fig. 7:**
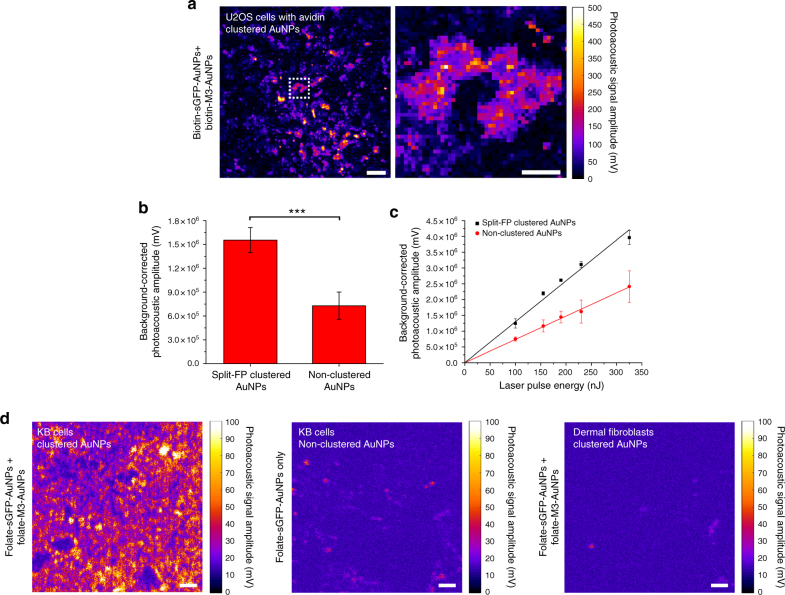
Photoacoustic imaging of in situ assembled split-FP AuNP clusters on cells. **a** Photoacoustic microscopy images of individual U2OS cells among a 100% confluent field after targeted clustering of biotin-M3-AuNPs and biotin-sGFP-AuNPs on cells that transiently express plasma membrane avidin biomarkers. Scale bars: 50 µm (left) and 20 µm (right). **b** Photoacoustic signal amplitudes from similar fields of U2OS cells targeted with both biotin-M3-AuNPs and biotin-sGFP-AuNPs (split-FP clustered) or with biotin-sGFP-AuNPs only (non-clustered). The mean photoacoustic signal amplitudes (±s.d.) were determined for *n* = 6 fields of views totaling 3 mm^2^ of cells at 100% confluence for each condition. ***p < 0.01, *t* test. **c** Photoacoustic signal amplitudes from targeted U2OS cells at increasing laser excitation energy. The mean photoacoustic signal amplitudes (±s.d.) were determined for *n* = 4 fields of views with cells at 100% confluence for each condition. Lines represent linear regression fit of the data. **d** Photoacoustic microscopy images of carcinoma KB cells targeted with both folate-sGFP-AuNPs and folate-M3-AuNPs (clustered AuNPs, left), of carcinoma KB cells targeted with folate-sGFP-AuNPs only (non-clustered AuNPs, middle) and of primary dermal fibroblasts targeted with both folate-sGFP-AuNPs and folate-M3-AuNPs (clustered AuNPs, right). Scale bars: 50 µm

Once co-targeted to U2OS cells expressing the avidin biomarkers, the self-assembly of 40 nm biotinylated M3-AuNPs and sGFP-AuNPs into nanoclusters resulted in strong photoacoustic signal amplifications and individual cells could be specifically imaged by photoacoustic microscopy (Fig. 7a). On targeted cells, the photoacoustic signal amplitude of the assembled clusters was about twice that of cells labeled with biotin-sGFP-AuNPs only (Fig. 7b), consistent with the fact that clustered AuNPs provide enhanced photoacoustic signals compared to individual AuNPs^71^ because thermal interfacial conductivity increases significantly within closely spaced AuNPs, notably along NP chains^72^. For both clustered and non-clustered biotinylated AuNPs, the photoacoustic signals scaled linearly with the range of laser excitation energy tested (Fig. 7c) This indicates that the enhanced photoacoustic signal amplitude primarily stems from the formation of discrete AuNPs clusters on targeted U2OS cells rather than from endocytosed AuNPs, which often display non-linear photoacoustic responses with increasing excitation intensities^33^.

In FR-overexpressing KB cells, the formation of split-FP assembled AuNP clusters after co-targeting both 40 nm folate-M3-AuNPs and folate-sGFP-AuNPs also resulted in easily detectable photoacoustic signals (Fig. 7d), which now scaled non-linearly with the different laser excitation energy tested (Supplementary Fig. 27). This non-linear response is consistent with the expected endocytosis of FR-targeted AuNPs clusters inside KB cells as mentioned above and as already implied by the detection of the neutral GFP chromophore SERS mode in confocal Raman microscopy images (Fig. 6b). In comparison, KB cells targeted with only folate-sGFP-AuNPs displayed weaker photoacoustic signals (Fig. 7d), which we attributed to the endocytosis of non-clustered folate-sGFP-AuNPs. Thus, despite cellular endocytosis, the assembly of folate-functionalized AuNP clusters in targeted KB cells clearly provides enhanced photoacoustic detections compared to non-clustered AuNPs.

To verify that such enhancements of photoacoustic signals are specifically correlated with the over-expression of FR biomarkers in cancerous KB cells, primary dermal fibroblasts having normal FR expression levels were also imaged under the same conditions by photoacoustic microscopy. Despite the co-targeting of both folate-M3-AuNPs and folate-sGFP-AuNPs, photoacoustic signals were barely detectable on dermal fibroblasts (Fig. 7d). This observation is consistent with the absence of AuNP cluster formation as already determined by SERS imaging, and with the limited endocytosis of targeted folate-AuNPs in these primary cells because of their significantly lower FR expression levels compared to KB cells.

Thus, in addition to allowing activatable SERS detections, the in situ and split FP-assisted clustering of AuNPs yields enhanced photoacoustic signals compared to individual AuNPs on targeted cells and these amplifications facilitate the specific detection of pathogenic cells that overexpress cancer biomarkers. The self-assembled metal nanoclusters effectively serve as highly specific bimodal contrast agents for SERS and photoacoustic microscopy imaging with single-cell sensitivity.

## Discussion

We have shown that different split-FP fragments can be used to control the assembly of a variety of plasmonic NPs into photoacoustic and activatable SERS nanocluster probes with well-defined hot spots geometry, both in solution and directly in live cells. The irreversible reconstruction of fully folded FPs from complementary split FP domains appended to the surface of metal NPs provides stable nanoclusters having nanogap sizes determined by the precise positioning and transverse orientation of the FP β-barrel structure. While other high-affinity protein domains might be employed for similar NP assemblies, the autocatalytic formation of the FP chromophore provides a direct read out of the clustering process and serves as a highly sensitive Raman reporter for low background SERS imaging in complex biological environments with, potentially, single-molecule SERS detection sensitivity. As such, split FPs represent a versatile family of biocompatible scaffolds and Raman reporters, which can be engineered by site-directed mutagenesis to generate additional FP variants and photo-controllable FPs^73,74^ for spectral matching with the near-field optical spectra of metal NP clusters and for advanced Raman scattering applications such as resonance and photo-switchable SERS imaging.

Upon targeting to diffusing cell surface biomarkers, complementary AuNPs rapidly organized into linear clusters. This linear ordering of NPs at the plasma membrane together with the 2 nm gap size formed by the complemented FPs provide large SERS amplifications of the FP chromophore for high sensitivity and single-cell SERS imaging. They also induce strong signal amplifications for enhanced photoacoustic imaging. Although we primarily used off-resonance 532 nm excitation of the clusters in cells to directly compare their photonic responses with that of individual NPs, plasmon-plasmon couplings within 40 nm AuNP linear clusters induce a large shift of their maximum near-field enhancement wavelength towards 630 nm^47^. Imaging AuNPs clusters at this resonant excitation wavelength is expected to provide SERS detection of the GFP chromophore vibrational modes at the onset of the near-infrared spectrum with two orders of magnitude higher SERS enhancement factors^47^. Applying this split FP assembly strategy to other plasmonic nanomaterials such as nanoshells^75^ might provide even larger SERS enhancement factors for targeted Raman imaging and photoacoustic detection of cells or tissues further in the near-infrared.

As we have shown, targeting endogenous (FR) or non-endogenous (avidin) biomarkers overexpressed at the cell plasma membrane induces the activation of our nanoclusters and allows highly specific cell imaging by SERS and photoacoustic microscopy, including the selective detection of cancer cells over normal cells. Other endogenous surface markers overexpressed on cancer cells might be targeted with a similar strategy, for instance using complementary NPs functionalized with antibodies. Compared to pre-activated SERS probes, this dual NP assembly and in situ SERS activation should provide highly selective detection of pathogenic cells over normal cells, notably if complementary NPs are targeted to two different overexpressed biomarkers. Indeed, because most cancer markers are also expressed on normal cells but at much lower levels, smart SERS and photoacoustic bimodal probes that switch on as a function of biomarker molecular density and diffusion, as shown here, can presumably reduce false positive detections, notably for in vivo imaging, where non-specific binding and uptake of nanoprobes by tissues are significant. We note that a good knowledge of the biomarker membrane mobility and potential lipid phase separation upon clustering is important to ensure an effective self-assembly of NP clusters on targeted cells. Further active control of the nanocluster assembly and, therefore, of SERS signals and photoacoustic enhancements, might be achieved by caging synthetic M3 peptides, for instance with photo-uncageable chemical moieties or with protease-responsive peptide sequences for uncaging and clustering within specific tissues in vivo.

Overall, the bottom-up assembly of colloidal metal NPs using split FP scaffolds as both molecular glue and Raman reporters addresses the long-standing issue of forming nanostructures having well-defined Raman hot-spots and that of site-specific SERS activation of nanoprobes in complex biological milieus. It provides a novel approach to remotely assemble nanocluster probes on biological targets for multimodal SERS and photoacoustic imaging with high sensitivity and selectivity in cells and, potentially, in vivo.

## Methods

### Expression and purification of sGFP

Plasmids encoding sGFP with a N-terminal 6xHis-tag, a GSS linker sequence, a thrombin cleavage site, a tetracysteine motif and a flexible GGSGG linker domain (Supplementary Fig. 1) were transformed in a BL21(DE3) *E. coli* strain for protein expression. 50 ml LB overnight starter culture (10 μg/ml kanamycin) was prepared with a transformed *E. coli* colony. 25 ml of the overnight culture was inoculated into 1 L LB (35 μg/ml kanamycin) and the culture was incubated in a shaker at 37 °C until OD_600_ reaches ~0.6. The culture was cooled down at room temperature for 20 min. After 1 mM IPTG induction, the culture was incubated overnight at 20 °C. Cells were harvested at 4000 g, for 30 min, at 4 °C. The cell pellet was washed with 20 ml ice cold PBS at 4000 g, for 30 min, at 4 °C. The cell pellet was suspended in TNG/imidazole buffer (100 mM Tris-HCl, 150 mM NaCl, 10% glycerol, 10 mM imidazole, pH 8.0). 1 × HALT protease inhibitor, 0.5 mM TCEP, 5 μl benzonase nuclease/g cell pellet, and 5 ml 1 × bugbuster/g cell pellet were added and incubated for 30 min at room temperature for cell lysis. The sample was centrifuged at 16000 × *g*, for 15 min, at 4 °C and the supernatant was collected. Since sGFP is expressed with a 6xHistag, Ni-resin beads were used to purify the sGFPs. TNG buffer with 10 mM imidazole and 150 mM imidazole were used as wash buffer and elution buffer, respectively. The sample was dialyzed against 1 l TN buffer (100 mM Tris-HCl, 150 mM NaCl, pH 8.0) for 1 h at 4 °C to remove imidazole. TN buffer was replaced and dialysis was continued overnight at 4 °C. A BCA protein assay was used to define the concentrations of the split-GFPs. Thrombin (15 U/mg protein) was used to remove 6xHistag from the proteins. Thrombin cleavage was performed for 20 min at room temperature in the presence of 1 mM TCEP. *p*-aminobenzamidine beads were used to eliminate the thrombin after cleavage. The sample was dialyzed against 1 l TN buffer for 1 h at 4 °C to remove TCEP. TN buffer was replaced and dialysis was continued for overnight at 4 °C. 10% glycerol was added to the dialyzed samples before freezing. The proteins were frozen using liquid nitrogen and stored at −80 °C. Full-length super-folder GFP (_fl_GFP) and other split-FP variants were expressed and purified following this same protocol.

### Characterization of recombinant sGFP

The purity of sGFP was assessed by SDS gel electrophoresis and by direct comparison with a commercial sGFP (Sandia Biotech), before and after purification on Ni-resin beads and cleavage of the 6xHis-tag by thrombin. The recombinant protein was detected at ~26 KDa and was >90% pure, with a small percentage of unreduced dimers (~9%, Supplementary Fig. 2). After thrombin cleavage, the presence and the activity of the sGFP N-terminal tetracysteine motif were assessed by fluorescence labeling with ReAsh^76^. Gel electrophoresis was performed on 1% agarose gels after ReAsh labeling and/or complementation of sGFP with an excess of M3 peptides. Gels were scanned on a Biorad, Molecular Imager FX, with appropriate laser excitation and emission filters for GFP, ReAsh and GFP-to-ReAsh FRET detections (Supplementary Fig. 3). ReAsh binding and GFP-to-ReAsh FRET detection indicated that the tetracysteine motif is effectively present and active at the N-terminus of sGFP and that its activity is not influenced by the binding of complementary M3 fragments.

### Surface functionalization of NPs with sGFP and M3 fragments

To functionalize AuNPs with tetracysteine-sGFP, 0.5 μM sGFP was mixed with 300 μl citrate capped AuNPs (40 or 10 nm in diameter, optical density of 1.0, Sigma) in the presence of 0.5 μM thiolated-PEG-biotin (NanoCS) in NaPT buffer (8 mM NaH_2_PO_4_, 50 mM NaCl, 0.05% tween 20 at pH 8.0) and incubated overnight at room temperature. Excess protein was removed by multiple rounds of centrifugation at 7000 × *g* for 10 min. sGFP-coated AuNPs were re-suspended in NaPT buffer before use. In addtion to the surface modification of AuNPs with sGFP, sYFP, and sCFP expressing the same N-terminal tetracysteine motif were used to functionalize AuNPs. The expression, the purification and the coating procedure for these split FP variants was the same as for sGFP. As observed for sGFP, the surface coating of AuNPs with sYFP or sCFP resulted in stabilization of the colloidal NPs which were monodispersed and did not show signs of aggregation when analyzed by DLS or TEM (Supplementary Fig. 5 and 6).

To coat AuNPs with the M3 peptide fragment, synthetic and cysteine-modified M3 peptides (C-acplinker-GSGGGSTSRDHMVLHEYVNAAGIT, Lifetein LLC, purity >75%) were used. 200 μM of M3 peptide and 20 μM thiolated-PEG-biotin were mixed with 300 μl AuNPs (40, 20, or 10 nm in diameter, optical density of 1.0, Sigma) in NaPT buffer. After overnight incubation at room temperature, excess peptide was removed by multiple rounds of centrifugation at 7000 × *g* for 10 min. M3-coated AuNPs were resuspended in NaPT buffer before use.

To functionalize oleylamine-stabilized AuNPs or AgNPs (10 nm diameter), two multicysteine synthetic peptides previously designed to bind to CdSe/ZnS quantum dots^49,77^ were used. These include (i) a small spacer-peptide with amino acid sequence KGSESGGSESGFCCFCCFCCF that provides hydrophilicity and makes space for (ii) a M3 peptide sequence FCCFCCFCCFGGSESG-(dPEG6)-GSGGGSTSRDHMVLHEYVNAAGIT that is more hydrophobic and provides reactivity to sGFP on AuNPs. Peptides dissolved in DMSO were rapidly mixed with AuNPs or AgNPs in toluene, and a few microliters of tetramethylammonium hydroxide (Sigma-Aldrich) was immediately added (i) to trigger the formation of cysteine thiolates anion in the peptides, (ii) to remove the hydrophobic surfactant from the surface of AuNPs or AgNPs, and (iii) to drive the binding of the peptides on NPs^77^. A slurry pellet was obtained after a vigorous shaking step. The supernatant was carefully removed and the pellet was dissolved in DMSO before a slow buffer exchange step on a G-10 column (Harvard Apparatus) equilibrated with distilled water. The eluate was extensively dialyzed against a NaP buffer in cellulose ester dialysis membranes (Spectra/Por® Biotech, 100 kDa MWCO, Spectrum Laboratories, Inc.) to remove non-reacted peptides.

### Assessing the presence of sGFP on AuNPs

To confirm the binding of sGFP at the surface of AuNPs we first compared the colloidal stability of citrate-stabilized bare AuNPs with that of sGFP-coated AuNPs. AuNPs were run on 0.8% agarose gels with 1 × TAE buffer pH 8. Under these conditions, citrate-stabilized AuNPs rapidly aggregate and do not migrate in the gel, but AuNPs coated with sGFP or with _fl_GFP are stabilized against aggregation and migrate as narrow bands (Supplementary Fig. 4), indicating the effective presence of the proteins at the surface. In a more direct approach, we also detected the presence of sGFP on AuNPs by immuno-blot assays against GFP directly on the NPs after protein coating and purification (Fig. 1c). For blotting, a strip of transfer membrane was first rinsed with methanol, water, and TBS buffer (20 mM Tris-HCl, 150 mM NaCl, pH 7.4). AuNP samples were then spotted on a PVDF membrane (Biorad) and further incubated until they were fully absorbed in the membrane but not dried out. The membrane was then rinsed with TBS buffer and was incubated at room temperature for 30 min in blocking buffer, 5% dry milk in TBST (0.05% tween-20 in TBS buffer). The blocking buffer was removed and the membrane was washed three times for 5 min with TBST buffer. A polyclonal rabbit anti-GFP primary antibody (ThermoFisher A-6455, 1:2000 dilution) was applied for 1 h at room temperature in blocking buffer. The membrane was washed three times in TBST buffer for 5 min. A goat anti-rabbit HRP secondary antibody (ThermoFisher 32260, 1:4000 dilution) was then applied for 1 h at room temperature in blocking buffer. The membrane was further washed three times with TBST buffer for 5 min and SuperSignal West Pico chemiluminescent substrate (ThermoFisher, 34080) was applied to the membrane. A Biorad Chemidoc system was used for chemiluminescence detection with 10 min of exposure time. As seen in Fig. 1c, bare AuNPs (citrate-stabilized) do not induce an immuno-reaction when targeted by anti-GFP antibodies, but both _fl_GFP and sGFP-coated AuNPs do, which provides direct evidence of the stable anchoring of both proteins at the surface of the metal NPs.

### Colloidal characterization of AuNPs

The mean hydrodynamic diameter (±s.d.) of AuNPs was measured on a dynamic light scattering (DLS) instrument (Wyatt Technology, DynaPro Titan) using 10 s acquisitions and a series of 30 repetitive measurements at 25 °C for each sample. Zeta potentials for AuNPs in NaPT buffer pH 8.0, were determined by averaging six independent measurements on a Malvern Zetasizer Nano ZS instrument equipped with a HeNe laser operating at 632.8 nm and a scattering detector positioned at 173° (Nanocomposix). For electron microscopy, AuNPs were intentionally deposited at low density on TEM grids to prevent the formation of drying-mediated 2D NP assemblies. An aliquot 10 μl of AuNPs diluted in NaPT were dropped on parafilm and TEM grids (Ted Pella, Inc. Carbon Type-B, 200 mesh, Copper) were inversed on the 10 μl samples for 20 min. After deposition, the TEM grids were transferred on a drop of Milli-Q water to rinse off the buffer and avoid salt crystals. A JEOL Jem-2100 (LaB6) microscope operated at 200 kV was used for imaging and a Gatan software (GMS-3) was used for TEM image analyzes.

### Nanocluster assembly with split-FP fragments

AuNP clusters were formed by co-incubation of equivalent amounts of sGFP-AuNPs (3.9 × 10^11^ NP/ml) and M3-AuNPs (3.9 × 10^11^ NP/ml) for 12 h in NaPT buffer at room temperature. This co-incubation time resulted in the formation of different sizes of nanocluster with more than 50% of all AuNPs being clustered (see Supplementary Fig. 11). Following co-incubation, AuNP samples were run at 120 V for 20 min in a 0.8% agarose gel equilibrated with 1 × TAE buffer. To purify the nanoclusters, the shifted/smear bands corresponding to AuNP clusters were cut and electro-eluted from the gel in cellulose ester dialysis membranes (Spectra/Por® Biotech, 100 kDa MWCO) in NaP buffer (8 mM NaH_2_PO_4_, 50 mM NaCl, pH 8.0). The purified nanoclusters were collected from the dialysis membrane and stored at 4 °C before further use. Alternatively, purification of some nanoclusters was done using multiple rounds of centrifugation at centrifugal forces adapted to the size and the sedimentation velocities of the AuNPs used in the assembly reaction. For instance, nanoclusters formed with 40 nm AuNPs reacted with 10 nm AuNPs were purified by a few rounds of centrifugation at 5000 g for 5 min. To compete with the assembly process, a large excess of free and non-cysteinilated M3 peptide (100 µM, RDHMVLHEYVNAAGIT, Lifetein LLC, purity >75%) was added during the co-incubation of sGFP-AuNPs and M3-AuNPs.

### Size distribution of nanoclusters and nanogap measurements

After 12 h co-incubation of sGFP-AuNPs with M3-AuNPs, unpurified samples were directly applied to TEM grids to perform statistical analyses on the formation of multimeric nanoclusters and to assess the size heterogeneity of the clusters. A total of 405 AuNP monomers and AuNP nanoclusters were evaluated from five independent experiments. In 53% of cases, AuNP nanoclusters with at least two AuNPs were observed. To define whether the formation of AuNP nanoclusters was random or effectively driven by complementation between split-FP fragments, we first compared the experimental distribution of AuNP nanocluster sizes with that expected for a random clustering process. In case of random clustering, a Poisson distribution of nanocluster sizes for a mean assembly efficiency of 53% is expected ($P(k) = \frac{{\lambda ^ke^{ - \lambda }}}{{k!}}$ with *λ* = 0.53). As shown in Supplementary Fig. 11, the experimental distribution is not well described by such a Poisson distribution. An F-test performed to determine if Poisson fit performed on the expected Poisson distribution (*λ* = 0.53) and on the actual cluster size distribution are significantly different from each other returned a *p*-value of 0.0177, indicating that both data sets are indeed significantly different from each other at the 95% confidence level (F-test: *p* < 0.05), and that the AuNP clustering process is not random.

To define which clustering kinetic regime leads to the observed distribution of cluster sizes, we plotted the experimental size distribution of AuNP clusters after normalization and log/log scale transformation^55^. In solution, the clustering of colloids can be described by a diffusion-limited aggregation (DLA) model or a reaction-limited aggregation (RLA) model^55,56^. For irreversible binding between colloids these models give markedly different aggregate’s dimension^55,56^ and very different cluster size distributions^55,56^. The DLA model leads (i) to a rapid formation of large, branched aggregates and (ii) to cluster size distributions that are characterized by a peaked distribution^55^. The RLA model leads (i) to the preponderant formation of smaller, more compact clusters due to the slower rates of interaction and (ii) to cluster size distributions that are best described by a power law distribution^55,56^. As shown in Fig. 2c, the nanocluster size distribution was well described by a power law fit, with a power coefficient of 1.7 ± 0.3 (coefficient ± s.d.), within the expect range for RLA processes (range: 1.5–2)^55,78^. This indicates that the formation of the AuNP nanoclusters is due to a reaction-limited aggregation as is expected for an assembly that is driven by the irreversible bimolecular complementation between the two split-FP fragments.

The size of the nanogap formed by the assembly of split FP fragment between AuNPs in nanoclusters was measured from TEM images. A Gatan software (GMS-3) was used to analyze the intensity profile of the AuNPs in images. The 50^th^ percentiles of the average of the maximum and minimum electron transmission intensities were used to define the edge of each AuNPs at the gap (Supplementary Fig. 12). 314 observations were used to analyze the gap sizes, which followed a Gaussian distribution with a mean of 2.1 nm and a standard deviation of the mean of 0.5 nm.

### Absorption spectroscopy of AuNP clusters

For absorption spectra measurements, sGFP-AuNPs (3.9 × 10^11^ NP/ml) and M3-AuNPs (3.9 × 10^11^ NP/ml) in NaPT buffer were mixed and spectra were acquired immediately following mixing (t = 0 min) and after t = 12 h of co-incubation at room temperature using a Perkin Elmer Lambda 950 spectrometer equipped with a 150 mm integrating sphere. Spectra were normalized at the surface plasmon absorption maximum of AuNP monomers (528 nm) and the differential absorption spectrum between the two time points was plotted (Supplementary Fig. 17). The absorption of full length GFP at 5 μM was also measured and normalized for comparison with the differential spectra of AuNP clusters as shown in Supplementary Fig. 17.

### Raman spectroscopy

A Horiba, XploRA One microscope with a cuvette holder was used to take liquid Raman spectra of highly concentrated _fl_GFP (310 μM), _fl_YFP (232 μM), and _fl_CFP (363 μM) solutions (Supplementary Fig. 14). Samples were loaded in a quartz cuvette (Starna) and Raman spectra were acquired for 150 sec using 785 nm laser excitation at 3.33 mW/µm^2^. A TNG buffer blank correction was applied to each spectrum. The same instrument was used for liquid SERS spectra of sGFP-AuNPs, M3-AuNPs and AuNP clusters (3.9 × 10^11^ AuNPs/ml) or AgNP clusters (3.1 × 10^11^ AgNP/ml) and for liquid Raman spectra of _fl_GFP and NaPT or TNG buffers (Fig. 3b and Supplementary Fig. 16), but spectra were taken for 30 sec using 785 nm excitation at 20 mW/µm^2^ for AuNPs and _fl_GFP or taken for 30 sec using 532 nm excitation at 17 mW/µm^2^ for AuNP, _fl_GFP and AgNPs. Liquid Raman spectra of the anionic form of the _fl_GFP chromophore was measured in TNG at pH 8.0. That of its neutral form was measured after dialysis (Slide-A-Lyzer 20 K dialysis cassettes, Thermo Scientific) of the _fl_GFP overnight at 4 °C against sodium acetate buffer at pH 6.0. Liquid SERS spectra of sGFP-AuNPs, M3-AuNPs and AuNP or AgNP clusters were measured in NaPT buffer pH 8.0 (anionic) or after spinning down the NPs for 8 min at 8000 g and exchanging the NaPT buffer at pH 8.0 with the same volume of NaPT buffer at pH 6.0 (neutral).

For qualitative SERS measurements on 5 nm silver island plasmonic substrates, a Renishaw inVia confocal Raman Microscope was used. SERS spectra of _fl_GFP, sGFP and M3-complemented sGFP were acquired for 60 sec using a 532 nm laser excitation at 140 µW/µm^2^. SERS spectra of M3-AgNPs incubated with sGFP were acquired for 30 sec using a 532 nm laser excitation at 140 µW/µm^2^.

### Calculation of SERS enhancement factors

The experimental enhancement factor (EF) was calculated by computing the ratio of SERS signal from complemented GFP in AuNP clusters to Raman scattering signal from _fl_GFP for excitations at 785 nm or 532 nm using:

$${\mathrm{EF}} = \left( {{\mathrm{I}}_{{\mathrm{sers - GFP}}}\times\,{\mathrm{C}}_{{\mathrm{flGFP}}}} \right)\;/\;\left( {{\mathrm{I}}_{{\mathrm{Raman - flGFP}}}\times\,{\mathrm{C}}_{{\mathrm{GFP}}}} \right)$$

where I_sers-GFP_ is the SERS intensity at 1527 cm^−1^ for complemented GFP in AuNP clusters after 12 h co-incubation averaged over three independent experiments, I_Raman-flGFP_ is the Raman scattering intensity at 1527 cm^−1^ of a pure solution of _fl_GFP averaged over three independent measurements, and C_flGFP_ and C_GFP_ are the concentrations of _fl_GFP and of complemented GFP, respectively. C_GFP_ was calculated by taking into account the concentration of AuNPs in the reaction (C_AuNP_), the cluster size distribution after 12 h (dimers 28%, trimers 13%, tetramers 7%, pentamers 3% and hexamers 2%) and the expected number of complemented GFP (N_GFP_) for each type of cluster (1 per dimer, 2 per trimer, 3 per tetramer, 4 per pentamer and 5 per hexamer) as follows:

$$\begin{array}{ccccc}\\ {\mathrm{C}}_{{\mathrm{GFP}}} = & \left( {{\mathrm{C}}_{{\mathrm{AuNP}}}\times{\mathrm{0.28}}\times {\mathrm{N}}_{{\mathrm{GFP - dimer}}} + {\mathrm{C}}_{{\mathrm{AuNP}}}\times{\mathrm{0.13}}\times {\mathrm{N}}_{{\mathrm{GFP - trimer}}}} \right.\\ \\ & + {\mathrm{C}}_{{\mathrm{AuNP}}}\times{\mathrm{0.07}}\times{\mathrm{N}}_{{\mathrm{GFP - tetramer}}} + {\mathrm{C}}_{{\mathrm{AuNP}}}\times{\mathrm{0.03}}\\ \\ & \times {\mathrm{N}}_{{\mathrm{GFP - pentamer}}} + \left. {{\mathrm{C}}_{{\mathrm{AuNP}}}\times{\mathrm{0.02}} \times {\mathrm{N}}_{{\mathrm{GFP - hexamer}}}} \right)\\ \end{array}$$

### Streptavidin titration on biotin-AuNPs

To assess the in vitro binding of biotinylated and non-biotinylated M3-AuNPs and sGFP-AuNP to avidin, AuNPs were incubated with decreasing concentrations of streptavidin (Sigma, final concentrations: 3.33 μM, 0.83 μM, 0.42 μM, 0.17 μM, 41.67 nM, 25 nM, 16.67 nM, 3.33 nM) for 45 min at room temperature. Samples were run in 0.8% agarose gel at 50 V in TAE buffer pH 8.0 (Supplementary Fig. 20). The first lanes represent control samples without streptavidin. Shifted bands indicate that streptavidin only reacts with biotinylated M3-AuNPs or sGFP-AuNPs.

### Fluorescence and dark field imaging of biotin-AuNPs in cells

HeLa or U2OS cells (ATCC) were grown at 37 °C on borosilicate coverslips (Marienfeld, 25 mm diameter, #1.5 thickness) in DMEM media (Lonza) supplemented with 10% fetal calf serum (FCS). Cells were transiently transfected with cDNA coding for the transmembrane or the GPI avidin fusions for 24 h (XtremeGene, Roche). 4 h prior to incubation with biotinylated M3-AuNPs or sGFP-AuNPs, cells were starved in FCS-free DMEM at 37 °C to free the avidin fusions from biotin present in the FCS supplement and to avoid competition with the biotinylated NPs. Starved cells were incubated with sGFP-AuNPs or M3-AuNPs separately for 1 h at 37 °C at a concentration of 0.57 × 10^10^ AuNPs/ml (dark field imaging) or 1.43 × 10^11^ AuNPs/ml (total internal refection imaging).

For correlated dark field imaging of targeted AuNPs and fluorescence imaging of residual avidin fusions at the cell plasma membrane, cells were additionally incubated with 2 µM of biotin-Alexa594 for 10 min at 37 °C before multiple rinses in PBS (Lonza) and cell fixation in 4% paraformaldehyde for 15 min. Microscopy imaging was done in PBS on an inverted Nikon Eclipse Ti-E microscope equipped with a Plan Fluor ELWD x40 objective (Nikon), a condenser lens, a mercury lamp, appropriate optical filters for Alexa594 imaging (Exc:562DF40, Dichroic: 593-Di03 and Em: 641DF75, Semrock) and an Ixon Ultra EMCCD camera (Andor).

Total internal refection fluorescence (TIRF) microscopy of M3-AuNPs or sGFP-AuNPs targeted at the bottom membrane of expressing cells was performed on the same inverted Nikon Eclipse Ti-E microscope equipped with a x100, 1.49 NA objective, TIRF optics, a 561 nm laser line, appropriate optical filters (Exc: ZET405/488/561/647 × , Dichroic: ZT405/488/561/647 and Em: 600DF50, Chroma) and an Ixon Ultra EMCCD camera (Andor). Images were acquired at 100 ms/frame.

### Scanning electron microscopy of AuNP nanoclusters in cells

Cells were grown at 37 °C on coverslips (Neuvitro, 15 mm diameter, #1 thickness) and transiently transfected with cDNA coding for the transmembrane and the GPI avidin fusions as described above. After 4 h starving in FCS-free DMEM, cells were incubated with 1.07 × 10^10^ NPs/ml of biotinylated M3-AuNPs and 1.07 × 10^10^ NPs/ml of biotinylated sGFP-AuNPs separately or simultaneously for 1 h at 37 °C. After a washing step with NaP buffer, ½ strength Karnovsky’s fixative was immediately applied for 1 h at room temperature to fix the cells. 0.1 M Cacodylate buffer was used to rinse the Karnovsky’s fixative and cells were treated with 2% osmium tetroxide for 30 min at room temperature for heavy metal staining. Increasing concentrations of ethanol were then applied for a gradual dehydration of the specimens, which were allowed to air-dry overnight at room temperature after application of hexamethyldisilizane. Images were acquired on a JEOL JSM-6390LV scanning electron microscope at 10 kV, with 5 mm working distance and a 30 nm spot size.

### Cell cytotoxicity assays

The cell cytotoxicity of bare AuNPs, biotinylated M3-AuNPs alone, biotinylated sGFP-AuNPs alone and AuNPs clusters formed by co-incubation of biotinylated M3-AuNPs+biotinylated sGFP-AuNPs on cells were compared to that of non-treated cells using a MTT [3-(4,5-dimethylthiazol-2-yl)-2,5-diphenyltetrazolium bromide] cell proliferation assays. In brief, 3500 U2OS cells/well were seeded on 96 well plates in DMEM + 10% FCS. After overnight attachment at 37 °C, cells were transiently co-transfected with cDNA coding for the transmembrane and the GPI avidin fusions for 24 h. Cells were then starved for 4 h in FCS-free DMEM, before application of biotinylated AuNPs at 1.07 × 10^10^ NPs/ml in 4 replicates per conditions. After 1 h incubation of AuNPs on cells, cells were washed five times in DMEM + 10% FCS and further incubated at 37 °C (except for t = 1 h) for 4, 12, 24 and 48 h. After incubation, cells were washed in FCS-free DMEM, before application of 100 µl of 12 mM MTT in FCS-free DMEM for 4 h at 37 °C. The MTT solution was then removed before addition of 50 µl DMSO for 10 min at 37 °C to dissolve the crystallized formazan and absorbance reading were acquired at 570 nm. Four additional wells were also prepared with only DMEM and MTT for background corrections. Data are presented as mean relative percentage of cell viability compared to non-treated cells ± standard error of the mean.

### SERS microscopy of biotin-AuNPs in avidin-expressing cells

For fixed cells, U2OS or HeLa cells were grown at 100% confluence on coverslips (Neuvitro, 15 mm diameter, #1 thickness), cotransfected with cDNA coding for the transmembrane and the GPI avidin fusions for 24 h (XtremeGene, Roche), starved and incubated with 1.07 × 10^10^ NPs/ml of biotinylated sGFP-AuNPs and 1.07 × 10^10^ NPs/ml of biotinylated M3-AuNPs separately or simultaneously as described above. Fixation was performed for 15 min at room temperature with 2% paraformaldehyde in NaP buffer, after washing the cells. The coverslips were then mounted on SiO_2_ wafers (300 nm oxide thickness, University Wafer) in NaP buffer pH 8.0 with 5% glycerol. A wide-field Rima Hyperspectral Imaging System (Photon Etc) equipped with a x100 objective, Bragg tunable filters and an EMCCD was used to map 130 × 130 μm^2^ areas of the cell samples with a 532 nm laser excitation at 200 µW/µm^2^. Each image was acquired for 60 sec with a 3 cm^−1^ spectral resolution. SERS spectra and SERS images of cells at specific Raman shifts were reconstructed with a PHysSpecV2 software (Photon Etc).

For live cells imaging, U2OS or HeLa cells were grown at 100% confluence directly on SiO_2_ wafer (300 nm oxide thickness, University Wafer), cotransfected with cDNA coding for the transmembrane and  the GPI avidin fusions for 24 h (XtremeGene, Roche), starved and incubated with biotinylated sGFP-AuNPs (1.07 × 10^10^ NPs/ml) and biotinylated M3-AuNPs (1.07 × 10^10^ NPs/ml) or with biotinylated sGFP-AgNPs (8.56 × 10^9^ NPs/ml) and biotinylated M3-AgNPs (8.56 × 10^9^ NPs/ml) simultaneously as described above. After 1 h incubation with NPs, cells were washed with PBS (8 mM NaH_2_PO_4_, 150 mM NaCl) at pH 8.0 and imaged in this buffer over a one-hour period. To stimulate early endocytosis, the PBS buffer was replaced by a hypotonic PBS buffer at pH 8.0 (8 mM NaH_2_PO_4_, 50 mM NaCl).

Confocal imaging was performed on a Renishaw inVia Raman Microscope equipped with a x40 water immersion objective. Cell samples were raster scanned in 1 μm step sizes using a 532 nm laser excitation at 6.73 mW/µm^2^ and using a cylindrical lens to spread the laser spot into a 70 μm × 1 µm line. At each scanning steps, signal integration was performed for 30 sec with 1.3 cm^−1^ spectral resolution. SERS spectra and SERS images of cells at specific Raman shifts were reconstructed using a home-made Matlab program.

### Fluorescence microscopy of folate receptor expression in cells

KB human carcinoma cells (ATCC) which overexpress FRs and immortalized human primary dermal fibroblasts (a kind gift from Dr. Howard Worman, Columbia University) which have normal FR expression levels were maintained in DMEM supplemented with 10% FCS at 37 °C. After a brief wash in 37 °C PBS (Lonza), cells grown on coverslips were fixed with 4% paraformaldehyde in PBS for 15 min at room temperature. Coverslips were then washed three times with PBS for 5 min and incubated with blocking buffer (0.6 mM BSA, 0.1% Tween-20 in PBS) for 30 min. Cells were then immunostained with a rabbit anti-FR alpha polyclonal antibody (Thermofisher Scientific, PA5-42004) at 1:100 ratio in blocking buffer for 1 h at room temperature. Cells were then washed three times with blocking buffer for 5 min before the addition of a goat anti-rabbit IgG Alexa Fluor 488 secondary antibody (Thermofisher Scientific, A-11034) at 1:300 ratio in blocking buffer for 1 h at room temperature. Cells were washed three times with blocking buffer for 5 min and with PBS for 5 min before being mounted in Fluoromount-G with DAPI (Electron Microscopy Sciences) and imaged by wide-field fluorescence imaging on an inverted Nikon Eclipse Ti-E microscope equipped with appropriate optical filters for DAPI and Alexa Fluor 488 detections.

### SERS microscopy of folate-AuNPs in KB and fibroblasts cells

Folate functionalized sGFP-AuNPs were produced by overnight incubation of 0.5 μM tetracysteine-sGFP with 300 μl of citrate capped AuNPs (40 nm in diameter, optical density of 1.0, Sigma)) in the presence of 0.5 μM thiolated-PEG_2000_-folate (NanoCS) and 0.5 μM thiolated-PEG_600_ (NanoCS) in NaPT buffer at room temperature. Excess sGFP and PEGs were removed by multiple rounds of centrifugation at 7000 g for 10 min, before resuspension of the folate-sGFP-AuNPs in NaPT buffer. Folate functionalized M3-AuNPs were produced by overnight incubation of 70 μM M3 peptide, 70 μM thiolated-PEG_2000_-folate and 70 μM thiolated-PEG_600_ with 300 μl AuNPs (40 nm in diameter, optical density of 1.0, Sigma) in NaPT buffer. After overnight incubation at room temperature, excess peptide was removed by multiple rounds of centrifugation at 7000 g for 10 min before resuspension of the folate-M3-AuNPs in NaPT buffer.

KB cells or dermal fibroblasts, cultured for at least 3 weeks in folate-free DMEM (FF-DMEM, Sigma D2429) + 10% FCS, were grown on SiO_2_ wafers and starved for 1 h at 37 °C in FCS-free FF-DMEM before incubation with both folate-sGFP-AuNPs (3 × 10^11^ NPs/ml) and folate-M3-AuNPs (3 × 10^11^ NPs/ml).

To prevent excessive endocytosis of the AuNPs after binding to FR at the plasma membrane of cells and allow the formation of NP clusters, both folate-sGFP-AuNPs and folate-M3-AuNPs were incubated with cell for 1 h at 4 °C and then for 15 min at 37 °C. Cells were then washed with PBS (Lonza) and imaged in this buffer on a Renishaw inVia Raman microscope as described above for biotinylated AuNPs targeted to cell expressing avidin fusions. SERS spectra and SERS images of cells at specific Raman shifts were reconstructed using a home-made Matlab program.

### Photoacoustic imaging of AuNPs clusters on targeted cells

Cells were grown at 100% confluence on coverslips (Marienfeld, 25 mm diameter, #1.5 thickness), transfected, starved and incubated with 3 × 10^10^ NPs/ml of 40 nm biotinylated sGFP-AuNPs and 3 × 10^10^ NPs/ml of 40 nm biotinylated M3-AuNPs separately or simultaneously as described above. After a washing step with NaP buffer, cells were immediately fixed in ½ strength Karnovsky’s fixative for 1 h at room temperature. Fixed cells were immersed in PBS buffer for acoustic signal coupling. Imaging was performed on a custom photoacoustic microscope as previously described^27,79^. Briefly, the laser source is a diode-pumped solid-state Nd:YAG laser (Spot-10-200-532, Elforlight Ltd) with a wavelength of 532 nm and a pulse duration of 2 ns. The laser was first collimated by a lens system, then reflected by a 2D galvanometer (6230 H, Cambridge Technology), and, finally, focused on the sample by an achromatic objective (AC254-040-A, Thorlabs) with a focal length of 40 mm and a numerical aperture (NA) of 0.1. Excited acoustic signals were captured by a custom ultrasonic transducer (center frequency: 35 MHz, 50% bandwidth at −6 dB), amplified by a low-noise amplifier (ZFL-500LN, Mini-Circuit), digitized by an A/D card (Cobra CompuScope CS22G8, GaGe), transferred to the computer, and, finally, reconstructed using a maximum amplitude projection (MAP) algorithm for visualization. For photoacoustic imaging and signal intensity measurements on U2OS cells at fixed laser excitation (Fig. 7a, b), a 532 nm laser pulse excitation energy of 130 nJ was used. For signal quantification, the means and standard deviations (s.d.) of the total photoacoustic amplitudes were calculated over a similar number of independent scanning areas (*n* = 6) totaling 3 mm^2^ of U2OS cells at 100% confluence. The mean total photoacoustic signal of transfected cells not incubated with AuNPs was used for background correction.

For photoacoustic imaging of KB cells and dermal fibroblasts, cells were grown on coverslips (Marienfeld, 25 mm diameter, #1.5 thickness) and incubated with 3 × 10^11^ NPs/ml of 40 nm folate-sGFP-AuNPs and 3 × 10^11^ NPs/ml of 40 nm folate-M3-AuNPs separately or simultaneously as described above. After a washing step with NaP buffer, cells were immediately fixed in ½ strength Karnovsky’s fixative for 1 h at room temperature and photoacoustic microscopy was performed as described above. For fixed laser excitation cell images (Fig. 7d), a 532 nm laser pulse excitation energy of 380 nJ was used. Additional cell images performed with lower 190 nJ excitation energy for KB cells and dermal fibroblasts and photoacoustic signal quantification as a function of laser pulse energy over a single-scanning area for KB cells are provided in Supplementary Fig. 26.

### Data availability

Matlab codes for the reconstruction of SERS images and the extraction of SERS spectra are available upon request by contacting Dr. Fabien Pinaud (pinaud@usc.edu). Matlab codes for the reconstruction of photoacoustic images are available upon request by contacting Dr. Chao Tian (ctian@med.umich.edu). These codes run on Matlab versions R2016b. All the other relevant data are available from the authors upon request.

## Electronic supplementary material

Supplementary Information

Descriptions of Additional Supplementary Files

Supplementary Movie
