# Supplementary material for: Cellular imaging by targeted assembly of hot-spot SERS and photoacoustic nanoprobes using split-fluorescent protein scaffolds
Source: Nat Commun. 2018 Feb 9;9:607. doi: 10.1038/s41467-018-03046-w (PMC5807522; doi:10.1038/s41467-018-03046-w)
Supplement: Supplementary file 1 — Supplementary Information [file 41467_2018_3046_MOESM1_ESM.pdf]

Supplementary Information

Cellular imaging by targeted assembly of hot-spot SERS and photoacoustic nanoprobes using split-fluorescent protein scaffolds

Köker et al.

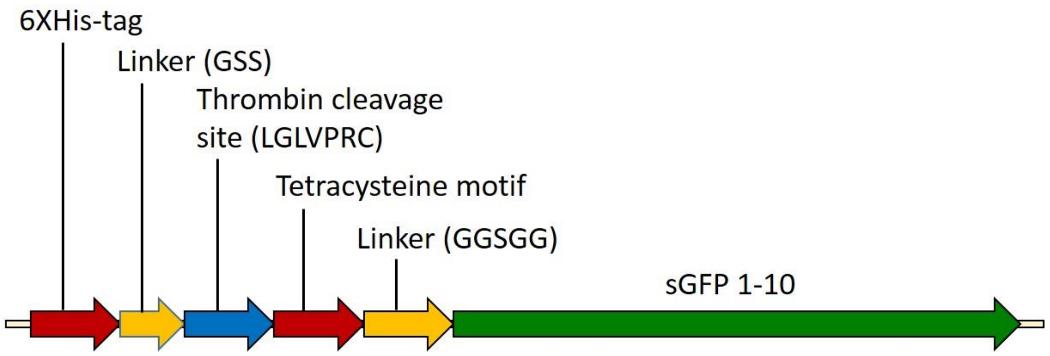

Supplementary Figure 1. Engineering sGFP for oriented binding on AuNPs. sGFP was expressed as a recombinant protein with: (i) a 6xHis-tag for purification of the protein, (ii) a GSS linker sequence, (iii) a thrombin cleavage site (LGLVPRC) to cut out the 6xHistag with thrombin after purification, (iv) a tetracysteine motif for oriented binding of sGFP at the surface of AuNPs, (v) a flexible GGSGG linker domain to limit conformation stiffness of the protein on AuNPs and (vi) the sGFP fragment coding sequence.

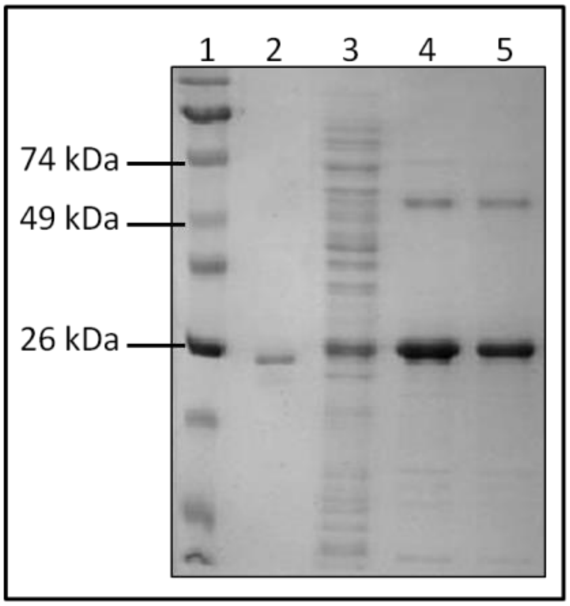

Supplementary Figure 2. SDS-PAGE gel electrophoresis characterization of the expression and the purification of the recombinant sGFP fragment. Lane 1: Molecular weight ladder. Lane 2: Commercial sGFP (~25KDa). Lane 3: Unpurified cell lysate. Lane 4: 6xHistag purified sGFP (~26KDa). Lane 5: Thrombin cleaved sGFP. A few higher molecular dimers of sGFP are observed (9%).

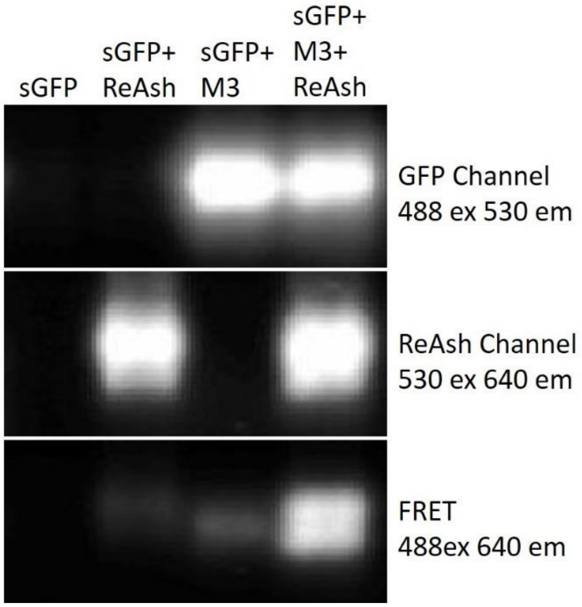

Supplementary Figure 3. Gel electrophoresis and ReAsh labeling to assess the presence and the activity of the tetracysteine motif at the N-terminus of the sGFP fragment. Lane 1: sGFP. Lane 2: ReAsh labeled sGFP. Lane 3: sGFP + M3 peptide fragment complementation. Lane 4: ReAsh labeled sGFP + M3 peptide fragment complementation. Notice that intramolecular FRET between complemented GFP and ReAsh bound to the tetracysteine motif is observed in lane 4.

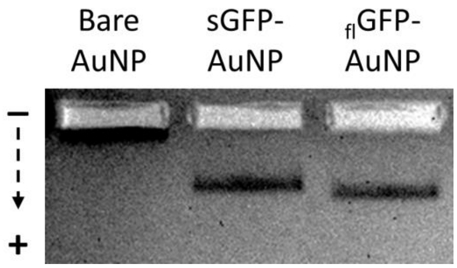

Supplementary Figure 4. Agarose gel electrophoresis of AuNPs. Lane 1: bare, citrate-stabilized AuNPs. Lane 2: sGFP-AuNPs. Lane 3: full-length super-folder GFP-coated AuNPs. The direction of the electric field applied is indicated by the arrow. Notice that bare AuNPs are unstable under these electrophoresis conditions and aggregate shortly after entering the gel.

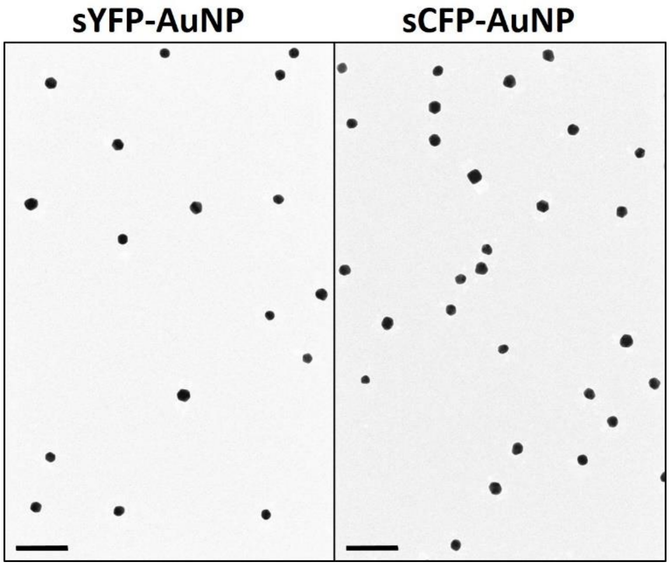

Supplementary Figure 5. TEM images of AuNPs functionalized with split-fluorescent protein variants sYFP and sCFP. Scale bars: 200 nm.

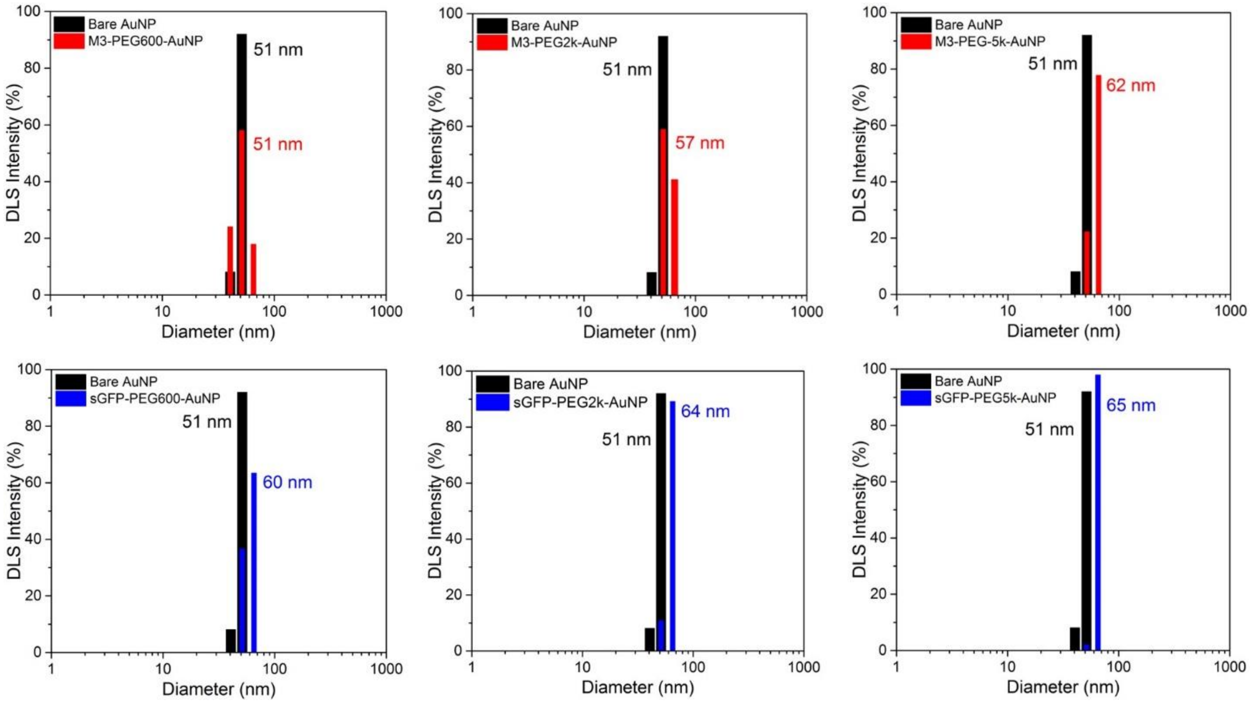

Supplementary Figure 6. Dynamic light scattering characterization of AuNPs. The size distribution of bare AuNPs is compared to AuNPs functionalized with sGFP or M3 peptide fragments and with different sizes of biotin-PEG moieties (5000 Da, 2000 Da, and 600 Da).

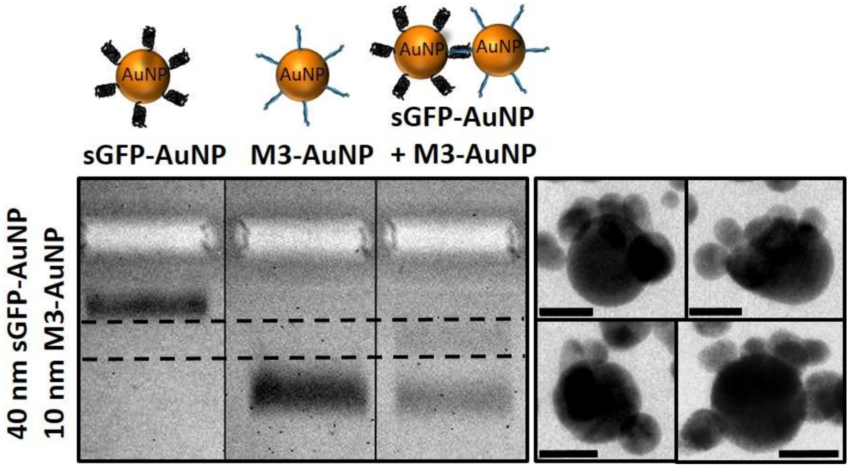

Supplementary Figure 7. Formation of AuNP clusters by the assembly of FP fragments. Agarose gel electrophoresis of 40 nm citrate-coated AuNPs functionalized with sGFP and co-incubated with 10 nm oleic acid-coated AuNPs functionalized with M3 peptides. TEM images of some of the nanoclusters observed after gel extraction of the gel band corresponding to the nanoclusters (dash lines) are shown. Scale bar: 20 nm.

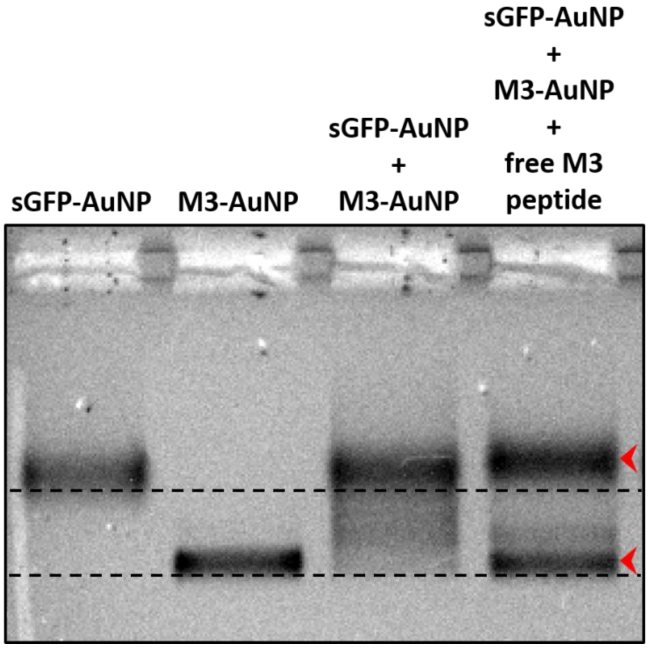

Supplementary Figure 8. Gel electrophoresis competition assay of the formation of AuNPs nanoclusters using an excess of free and non-thiolated M3 peptide fragment. Upon 12 hours co-incubation of 10 nm sGFP-AuNPs (lane 1) with 40 nm M3-AuNPs (lane 2), a typical smeared band of AuNP nanoclusters is observed together with the disappearance of the M3-AuNP band (lane 3). If a large excess (100 µM) of free, non-cysteinilated M3 peptide fragment is added at the beginning of the co-incubation, little to no smearing is observed and both sGFP-AuNP and M3-AuNP bands remain intact (red arrow heads). This indicates that the formation of AuNP clusters is solely driven by the self-assembly of split fluorescent protein fragments appended at the surface of the nanoparticles.

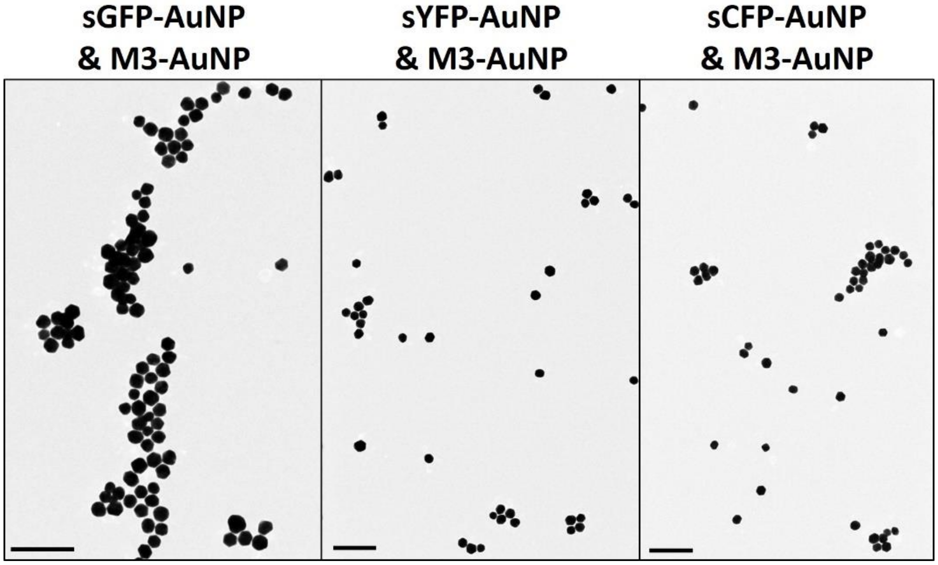

Supplementary Figure 9. TEM images of compact nanoclusters formed with sGFP or its spectral variants. sGFP-AuNPs, sYFP-AuNPs or sCFP-AuNPs were co-incubated with M3-AuNPs. Scale bar: 200 nm.

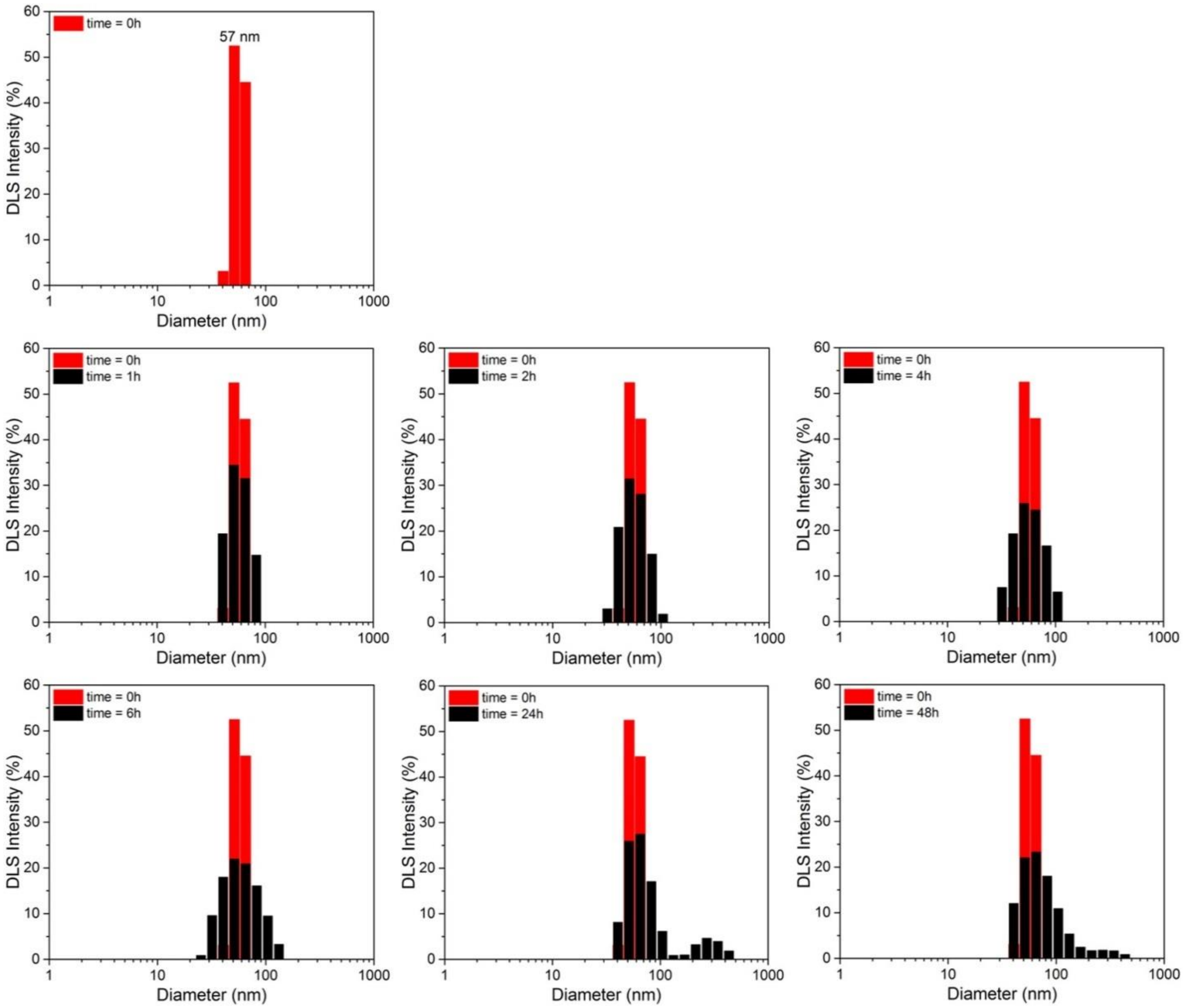

Supplementary Figure 10. Kinetic analysis of the formation of AuNP clusters in solution by dynamic light scattering. M3-AuNPs-PEG$_{600}$ (40 nm) were co-incubated with sGFP-AuNPs-PEG$_{600}$ (40 nm) in solution. The formation of AuNP clusters is assessed by measuring changes in the size distribution of the AuNPs using dynamic light scattering (DLS) measurements at different time intervals (1, 2, 4, 6, 24 and 48 hours) and comparing with their initial size distribution at time t=0 min.

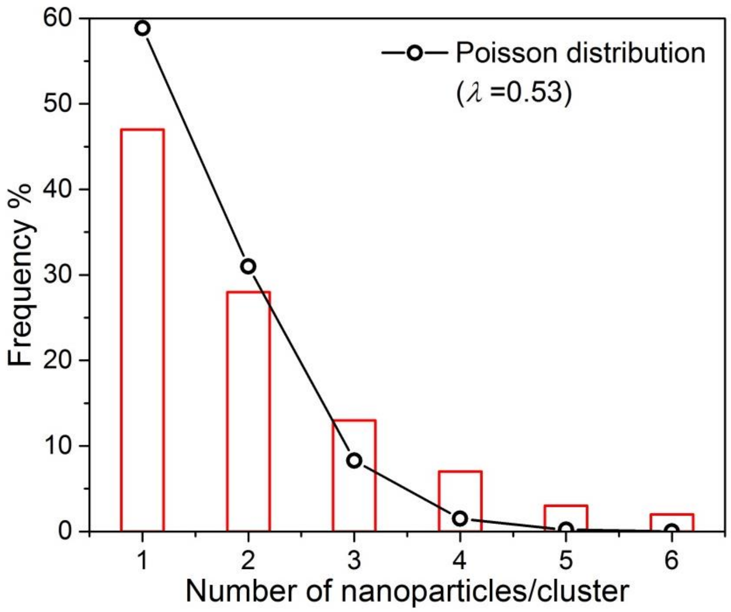

Supplementary Figure 11. Statistical size distribution of unpurified AuNP clusters observed by TEM. Clusters were formed by co-incubating sGFP-AuNPs (40 nm) with M3-AuNPs (40 nm) for 12 hours (red bars). The cluster size distributions were determined over five independent assembly experiments. 53% of all the AuNPs assemble into clusters containing at least two AuNPs. The distribution is compared to the expected Poisson distribution if the formation of the clusters was driven by random interactions between AuNPs (black line, $\lambda = 0.53$).

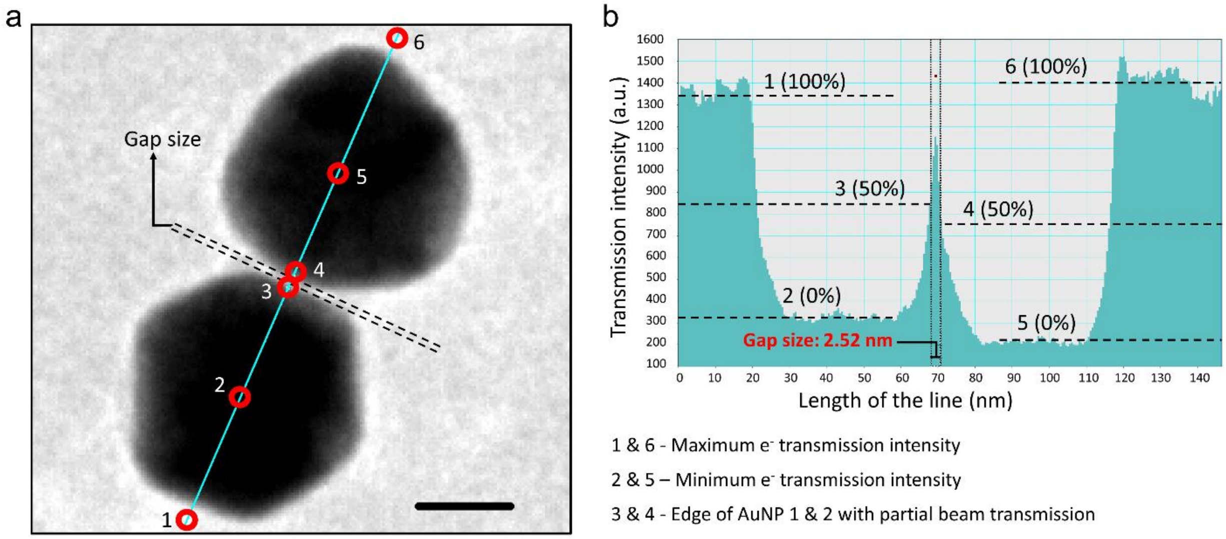

Supplementary Figure 12. Nanogap size measurement between clustered AuNPs. a) Example of a dimeric nanocluster formed with a 40 nm M3-AuNP and a 40 nm sGFP-AuNP. Scale bar: 20 nm. b) Electron transmission intensity profile along the cyan line on the dimeric nanocluster in (a). The average of the maximum and minimum electron transmission intensities is calculated from the intensity profile for each AuNP in a cluster. The 50% value is used to estimate the gap edge for both AuNPs and to measure the actual gap size.

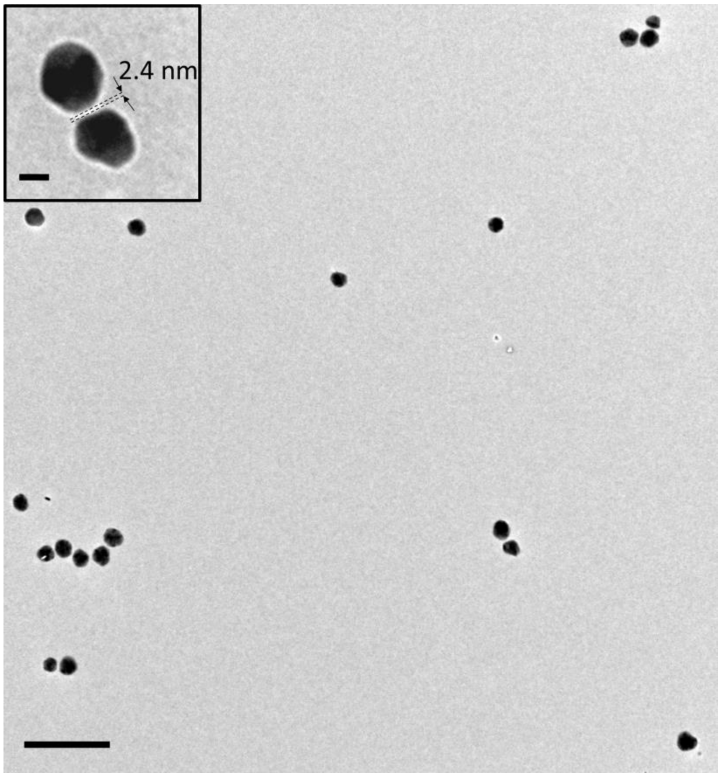

Supplementary Figure 13. TEM image of silver nanoclusters formed after co-incubation of 40 nm sGFP-AgNPs and 40 nm M3-AgNPs. Scale bar: 200 nm. Inset: nanogap size measurement of a dimeric AgNP cluster. Scale bar: 20 nm.

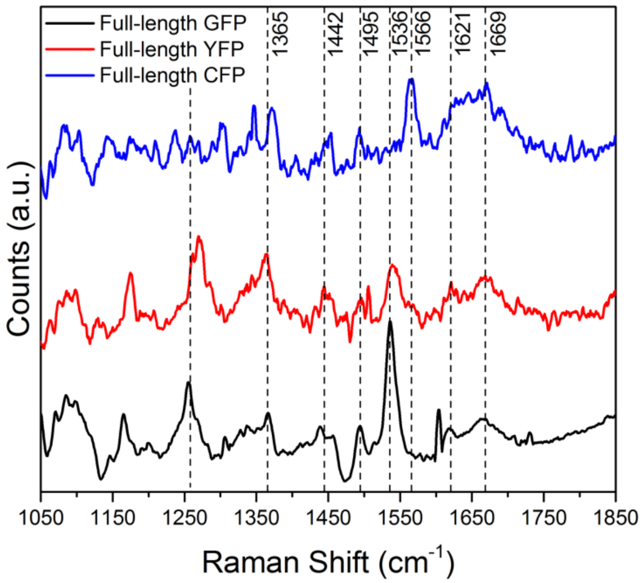

Raman shifts (cm^{-1})	Assignment
1210 – 1300	AmIII[a]
1365	δ(C_α-H)[a]
1442	δ(CH_2) and δ_{assym}(CH_3)[a]
1495	ν(C-C) and δ(CH) of aromatic amino acids[a]
1536 (anionic)	ν(C=C) exocyclic, imidazolinone[c]
1566 (neutral)	ν(C=C) exocyclic, imidazolinone[c]
1621	ν(C=C) exocyclic[c] and ν(C=C) and ν(C=N) of aromatic amino acids and histidine[a]
1669	AmI[a]

ν, stretching; δ, bending; [a]: protein and peptide Raman shifts, [c]: chromophore Raman shifts.

Supplementary Figure 14. Raman spectra of $_{fl}$FPs in aqueous buffer. For the $_{fl}$GFP (310 μM), $_{fl}$YFP (232 μM) and $_{fl}$CFP (363 μM) variants, the three main chromophore fingerprints at 1530 cm^{-1}, 1560 cm^{-1} and 1660 cm^{-1} are detected within ±10 cm^{-1} of their expected spectral positions, together with weaker vibrational bands of the chromophores and additional Raman bands attributed to the rest of the proteins. Detected Raman bands are assigned in reference to previously corresponding Raman shifts[1-6]. λ_{ex}: 785 nm, P_{ex}: 3.33 mW/μm^2, acquisition time: 150 seconds.

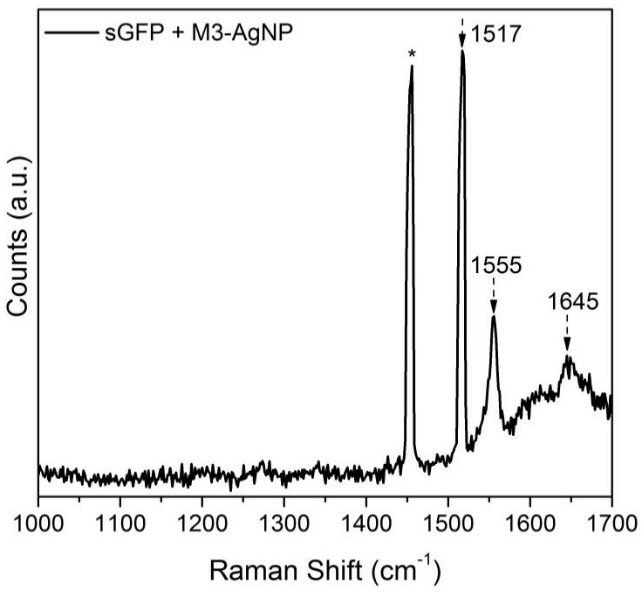

Supplementary Figure 15. SERS spectra of complemented GFP on silver nanoparticles and silver island plasmonic substrates. M3-coated silver nanoparticles were added onto 5 nm silver island plasmonic substrates shortly after co-incubation with the sGFP fragment. The typical GFP chromophore fingerprints are observed at 1517 cm^{-1}, 1555 cm^{-1} and 1645 cm^{-1}. *Assigned to the C-C-H in-plane deformation mode of the chromophore's phenol ring or the CH2/CH3 deformation mode of amino acids in complemented sGFP. λ_{ex}: 532 nm, P_{ex}: 140 μW/μm^2, acquisition time: 30 s.

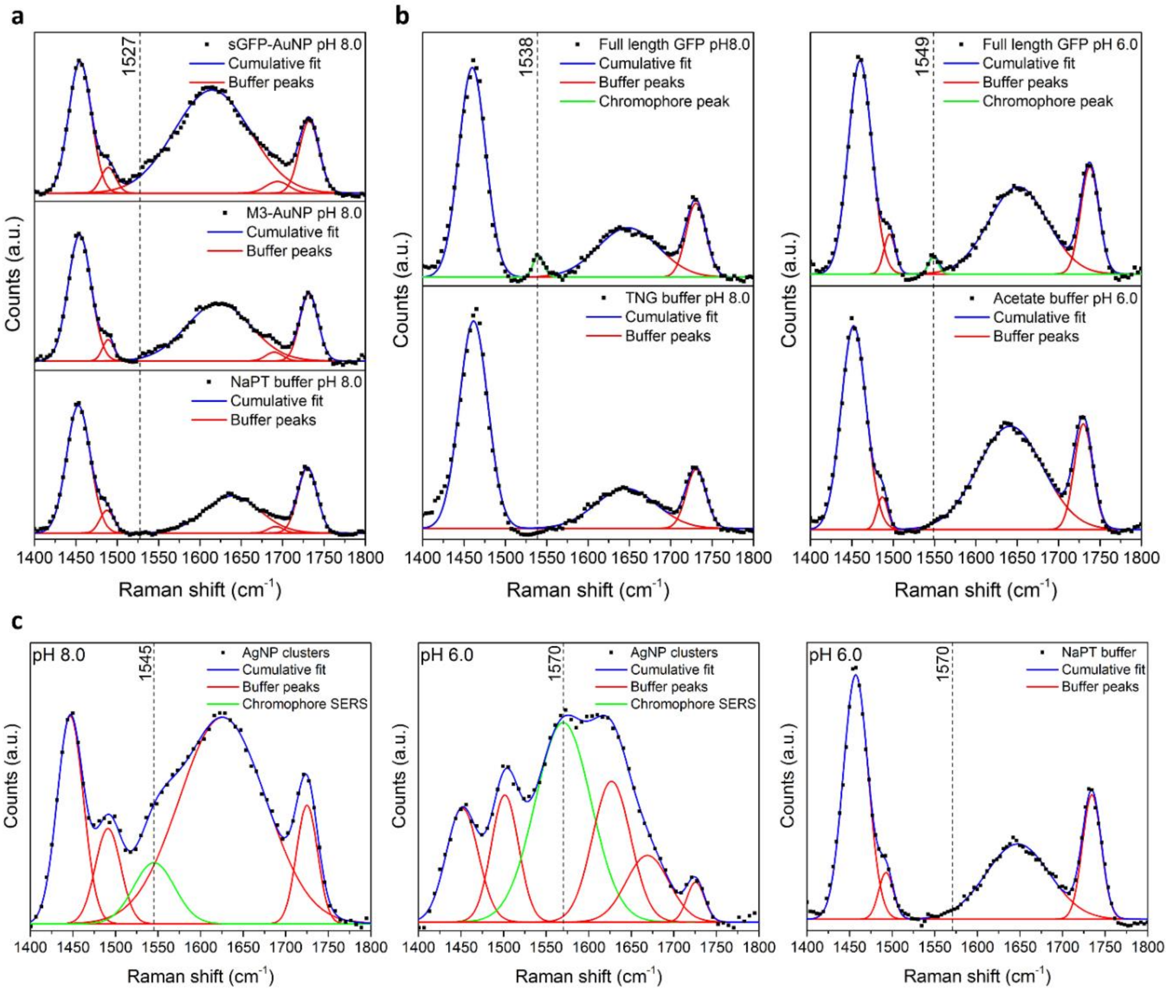

Supplementary Figure 16. SERS spectra of individual and clustered NPs in buffer solutions. a) SERS and Raman spectra of sGFP-AuNPs (top), M3-AuNPs (middle) and NaPT buffer (bottom) at pH 8.0. In the buffer alone and in the absence of AuNP clusters, the GFP chromophore vibrational signature is not detected. λ_{ex}: 785 nm, P_{ex}: 20 mW/μm^2, acquisition time: 30 s. b) Raman spectra of $_{fl}$GFP at pH 8.0 in TNG buffer (top left) and at pH 6.0 in acetate buffer (top right) and corresponding Raman spectra of pH 8.0 TNG buffer alone (bottom left) and pH 6.0 acetate buffer alone (bottom right). The 1538 cm^{-1} imidazolinone/exocyclic C=C mode of the anionic GFP chromophore in $_{fl}$GFP is detected among vibrational modes from the TNG buffer at pH 8.0. Change in pH induces the neutral form of the chromophore and a shift of the 1538 cm^{-1} mode toward 1549 cm^{-1}. λ_{ex}: 785 nm, P_{ex}: 20 mW/μm^2, acquisition time: 30 s. c) SERS spectra of assembled 40 nm M3-AgNP and sGFP-AgNP clusters at pH 8.0 (left) and pH 6.0 (center) and Raman spectra of NaPT buffer at pH 6.0 (right) obtained for an excitation at 532 nm to optimize SERS responses. The anionic imidazolinone/exocyclic C=C mode was detected at 1545 cm^{-1} in the SERS spectrum of AgNP clusters at pH 8.0. The neutral mode was detected at 1570 cm^{-1} in SERS spectra at pH 6.0. λ_{ex}: 532 nm, P_{ex}: 17 mW/μm^2, acquisition time: 30 s.

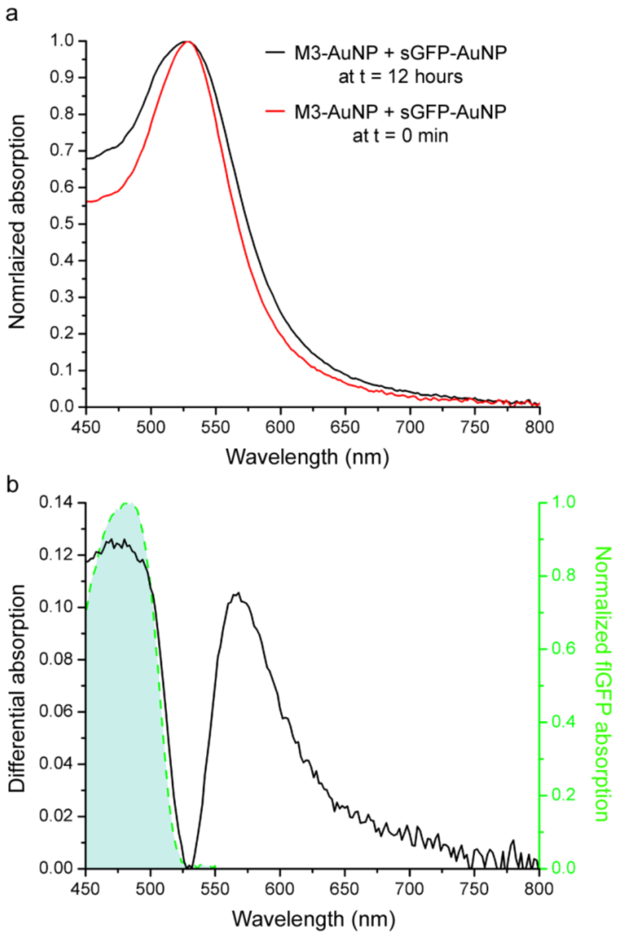

Supplementary Figure 17. Absorption spectra of AuNP clusters assembled by split-GFP fragments in an integrating sphere. a) Comparison of the absorption spectra for M3-AuNPs co-incubated with sGFP-AuNPs at t=0 min (AuNP monomers only) and at t=12 hours (AuNP clusters and residual non-assembled monomers). b) Differential absorption spectrum between spectra at t=0 min and t=12 hours in (a) showing the appearance of the plasmonically enhanced absorption band of complemented and mature GFP (at ~480 nm) and that of the longitudinal plasmon absorption modes of AuNP clusters above 550 nm (including dimers, trimers, tetramers, etc.). The measured absorption band of full length GFP ($_{fl}$GFP) is overlaid for comparison (green). We note that red-shifts of the longitudinal plasmon mode for AuNP cluster chains can be accompanied by small dipole-induced blue-shifts of the transverse plasmon mode, which are on the order of 0-15 nm for strongly coupled 2-6 AuNP chains (nanogaps of ~ 1 nm)[7]. The absorption band appearing at 480 nm in the split-FP assembled AuNP clusters is blue-shifted by 48 nm compared to the 528 nm resonance peaks of individual AuNPs. This shift being much larger than expected for 2 nm GFP-seeded nanogaps between AuNPs, it is not likely associated with a blue-shift of the transverse resonance of AuNPs dimers, trimers, etc., but stems from plasmonic enhancements of the optical absorption band of complemented and mature GFP in the clusters.

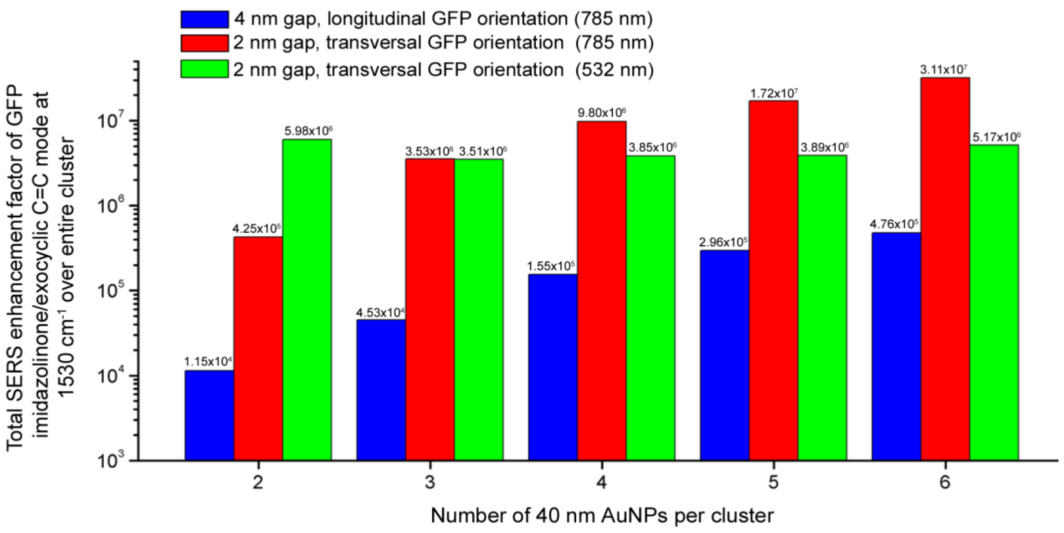

Supplementary Figure 18. Theoretical SERS enhancement factors calculated by finite-difference time-domain modeling. SERS enhancement factors of the GFP imidazolinone/exocyclic C=C mode at 1530 cm^{-1} are calculated for different sizes of AuNP clusters with GFP-seeded nanogap dimensions of 2 or 4 nm at 785 nm or 532 nm excitations. The clusters are modeled as linear chain assemblies of 40 nm AuNPs with n = 2, 3, 4, 5 or 6 AuNPs per chain. The total SERS enhancement factor per cluster is calculated by summing the integrated SERS enhancements within a 2x2x2 nm volume centered at the GFP position over each nanogap within each cluster (e.g. 1 nanogap for dimers, 2 nanogaps for trimers, 3 nanogaps for tetramers, etc.). The calculations are performed using:

$$EF_{SERS-GFP} \approx \sum \left|\frac{E(\omega_{exc})}{E_0(\omega_{exc})}\right|^2 \left|\frac{E(\omega_{vib-GFP})}{E_0(\omega_{vib-GFP})}\right|^2$$

where, for each nanogap, E is the amplitude of the local maximum electric field, E_0 is the amplitude of the input source field polarized parallel to the long axis of each cluster, here 1 V/m, $E(\omega_{exc})/E_0(\omega_{exc})$ is the enhanced field at 785 nm or 532 nm laser excitations and $E(\omega_{vib-GFP})/E_0(\omega_{vib-GFP})$ is the enhanced field at the 1530 cm^{-1} Stokes-shifted wavelength of the GFP chromophore imidazolinone/exocyclic C=C mode at 892 nm for a 785 nm excitation or at 579 nm for a 532 nm excitation. For more information about the modeling and the calculation methods, see Chung et al.[8]. To estimate the theoretical SERS enhancement factor from solutions of AuNP clusters, the cluster size distribution from **Supplementary Fig. 11** was taken into account (dimers 28%, trimers 13%, tetramers 7%, pentamers 3 % and hexamer 2%) and the total SERS enhancement for each type of cluster weighted by these percentages was summed. The SERS enhancement from non-clustered AuNPs (47%) was omitted, as it is comparatively negligible. For solutions with 2 nm gap AuNP clusters the 785 nm and 532 nm theoretical SERS enhancements are: $2.39x10^6$ fold and $2.61x10^6$ fold respectively. For solutions with 4 nm gap AuNP clusters the 785 nm theoretical SERS enhancement is $3.86x10^4$ fold. These represent upper limit electromagnetic enhancement factors as they are calculated for idealized and perfectly ordered clusters.

M3-AuNPs + sGFP-AuNPs clusters

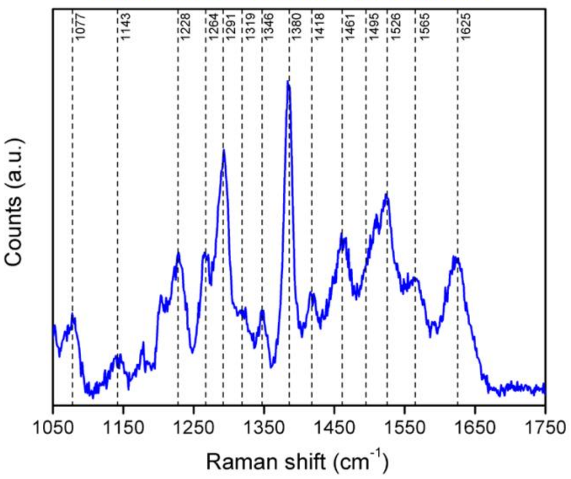

Raman shifts (cm^{-1})	Assignment
1077	v(C-N)[a] and v(C-C)[b,d]
1143	v(C-N)[a] and v(C-C)[a,d]
1228	AmIII (β-sheet)[a]
1264	AmIII (α-helix)[a]
1291	AmIII (α-helix)[a]
1319	$\delta(C_\alpha\text{-}H)$[a]
1346	$\delta(C_\alpha\text{-}H)$[a]
1380	$\rho_w(CH_2)$[b,d] and $\delta_{sym}(CH_3)$[a]
1418	$v_{sym}(COO^-)$[a], $\delta(CH_2)$[a,d] and $\delta_{assym}(CH_3)$[a]
1461	$\delta(CH_2)$[a,d] and $\delta_{assym}(CH_3)$[a]
1495	v(C-C) and δ(CH) of aromatic amino acids[a]
1526	v(C=C) exocyclic[c], imidazolinone[c] (anion)
1565	v(C=C) exocyclic[c], imidazolinone[c] (neutral)
1625	v(C=C) exocyclic[c] and v(C=C) and v(C=N) of aromatic amino acids and histidine[a]

sGFP-AuNPs only

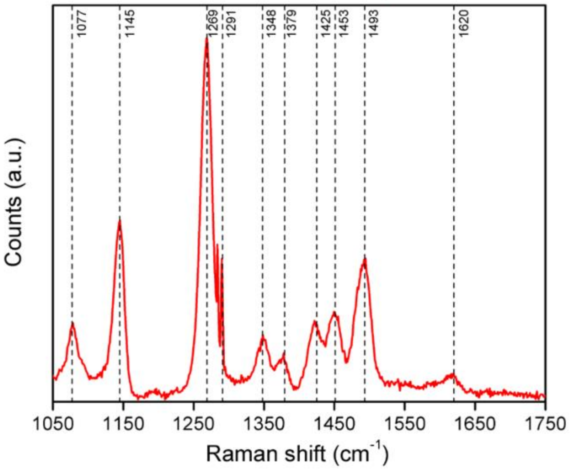

Raman shifts (cm^{-1})	Assignment
1077	v(C-N)[a] and v(C-C)[b,d]
1145	v(C-N)[a] and v(C-C)[a,d]
1269	AmIII (α-helix)[a]
1291	AmIII (α-helix)[a]
1348	$\delta(C_\alpha\text{-}H)$[a]
1379	$\rho_w(CH_2)$[b,d] and $\delta_{sym}(CH_3)$[a]
1425	$v_{sym}(COO^-)$[a], $\delta(CH_2)$[a,d] and $\delta_{assym}(CH_3)$[a]
1453	$\delta(CH_2)$[a,d] and $\delta_{assym}(CH_3)$[a]
1493	v(C-C) and δ(CH) of aromatic amino acids[a]
1620	v(C=C) and v(C=N) of aromatic amino acids and histidine[a]

M3-AuNPs only

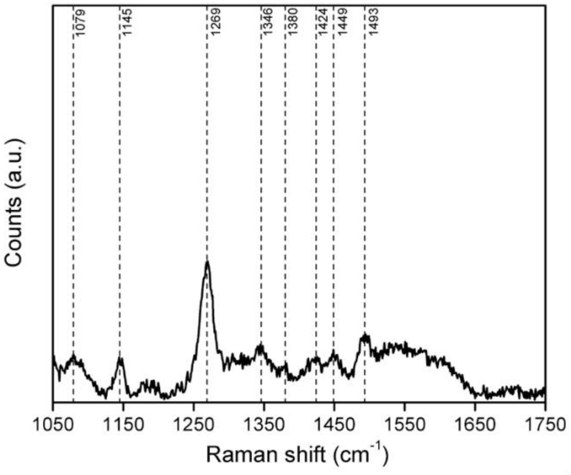

Raman shifts (cm^{-1})	Assignment
1079	v(C-N)[a] and v(C-C)[b,d]
1145	v(C-N)[a] and v(C-C)[a,d]
1269	AmIII (α-helix)[a]
1346	$\delta(C_\alpha\text{-}H)$[a]
1380	$\rho_w(CH_2)$[b,d] and $\delta_{sym}(CH_3)$[a]
1424	$v_{sym}(COO^-)$[a], $\delta(CH_2)$[a,d] and $\delta_{assym}(CH_3)$[a]
1449	$\delta(CH_2)$[a,d] and $\delta_{assym}(CH_3)$[a]
1493	v(C-C) and δ(CH) of aromatic amino acids[a]
1500-1650	background broadening from PEG_{600} brush[d]

v: stretching; δ: bending; ρ_w: wagging; [a]: protein and peptide Raman shifts; [b]:biotin Raman shifts; [c]: chromophore Raman shifts; [d]: PEG Raman shifts.

Supplementary Figure 19. SERS spectra of intentionally aggregated AuNPs and AuNP clusters. A 50 µl drop of sGFP-AuNPs, M3-AuNPs, or AuNP clusters assembled for 12 hours was deposited on a SiO_2 wafer and intentionally aggregated following the evaporation created by the laser. AmIII bands and CH_2/CH_3 scissor bending from sGFP and M3 peptides are observed from all samples because of the induced aggregation. The GFP chromophore Raman fingerprints are observed for the aggregated clusters despite the presence of protein and peptide Raman shifts. Raman bands are assigned in reference to previously corresponding Raman shifts[1-6]. λ_{ex}: 785 nm, P_{ex}: 33 mW/µm^2, acquisition time: 60 s.

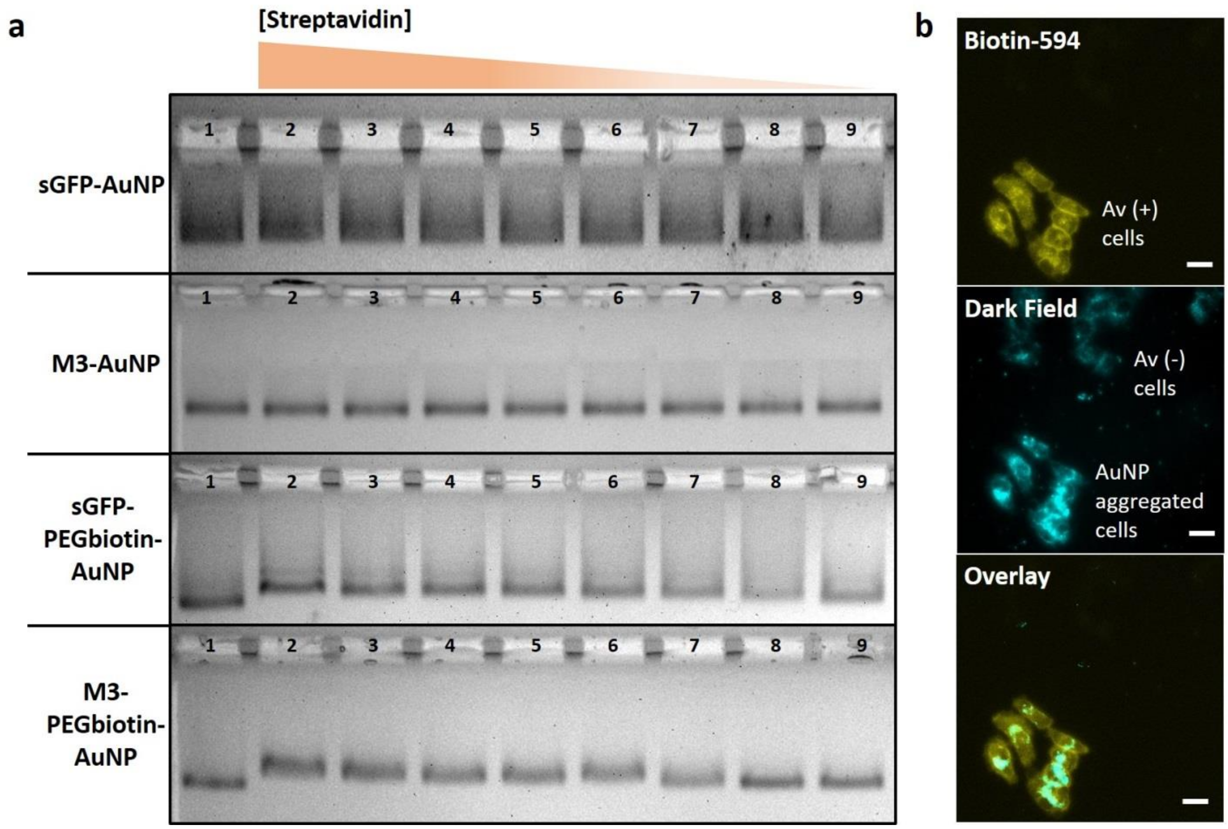

Supplementary Figure 20. Reactivity of biotinylated AuNPs with streptavidin and specific targeting at the plasma membrane of live cells expressing extracellular avidin fusion proteins. a) 0.8% agarose gel shift assay after incubation of sGFP-AuNPs or M3-AuNPs with decreasing concentrations of streptavidin (3.33 µM, 0.83 µM, 0.42 µM, 0.17 µM, 41.67 nM, 25 nM, 16.67 nM, 3.33 nM) for 45 min at room temperature. While no shift is observed for non-biotinylated M3-AuNPs and sGFP-AuNPs, discrete shifts are seen for the biotinylated NPs at high streptavidin concentrations. This indicates that surface attached biotin-PEG moieties effectively react with streptavidin despite the additional presence of M3 peptide or sGFP fragments. b) Top: Fluorescence wide-field microscopy image of fixed HeLa cells expressing the membrane avidin fusions and labeled with biotin-Alexa594 fluorophore (yellow). Middle: Dark field microscopy image of the cells (cyan). Bottom: Overlay of fluorescence and dark field images. Expressing cell targeted with biotinylated sGFP-AuNPs display larger dark field signals than non-targeted cells, which only show weak dark-field signals due to scattered light from membranes and internal organelles. Scale bars: 20µm.

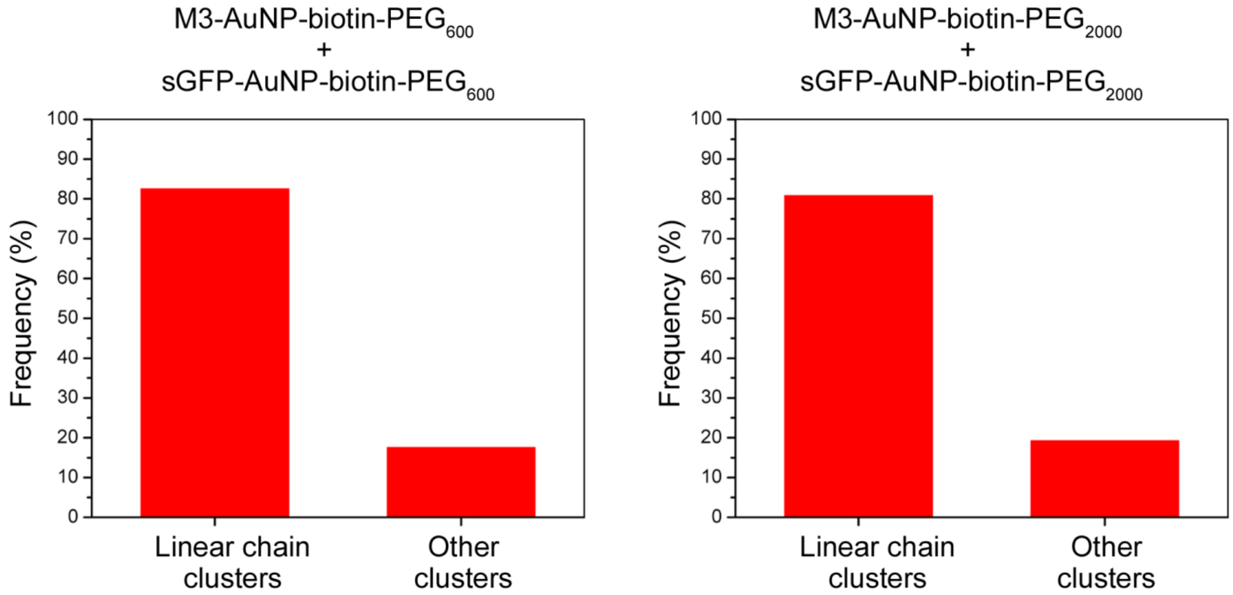

Supplementary Figure 21. Quantification of linear chain AuNP clusters at the plasma membrane of cells. The frequency of linear nanoclusters is quantified for M3-AuNP-biotin-PEG_{600} co-targeted with sGFP-AuNP-biotin-PEG_{600} (left) and for M3-AuNP-biotin-PEG_{2000} co-targeted with sGFP-AuNP-biotin-PEG_{2000} (right).

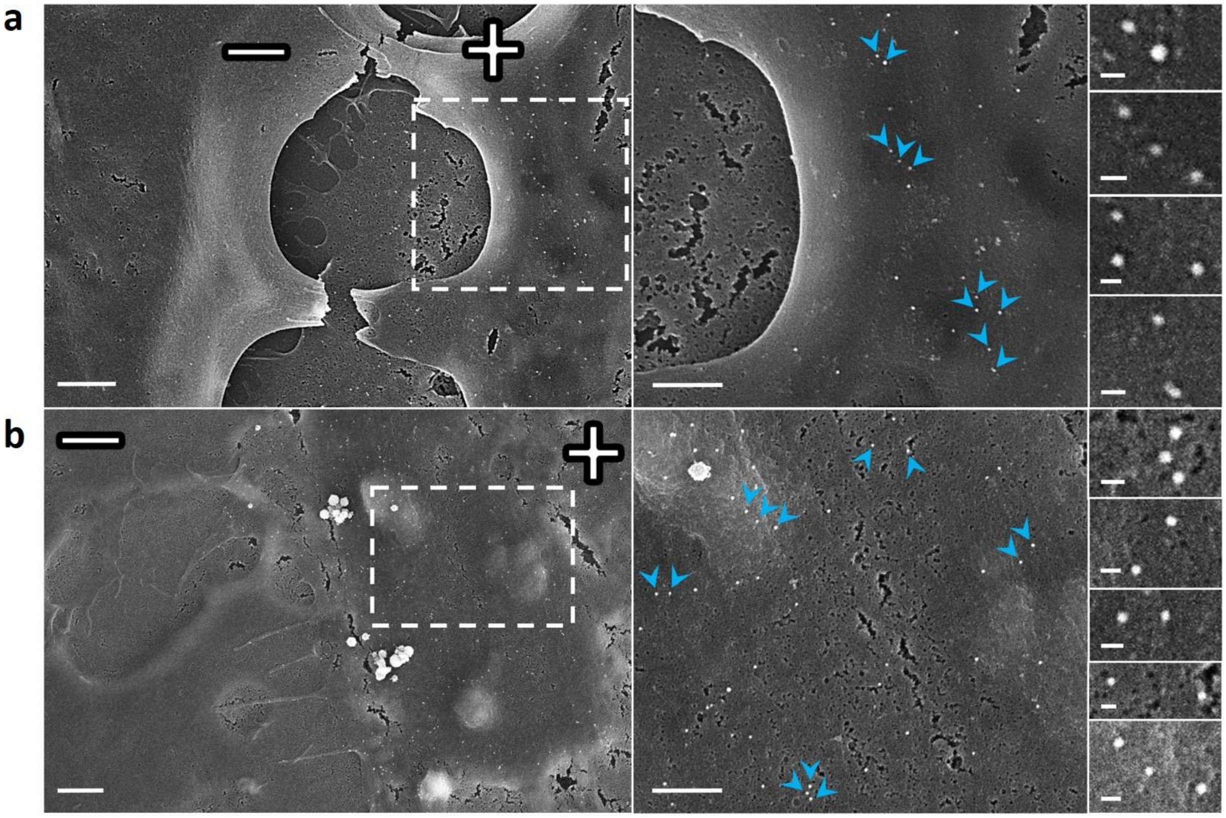

Supplementary Figure 22. SEM images of U2OS cells expressing both avidin fusions and co-targeted with AuNPs lacking surface biotin. a) Co-targeting of M3-AuNPs without biotin together with sGFP-AuNP-biotin-PEG_{600}. b) Co-targeting of sGFP-AuNPs without biotin together with M3-AuNP-biotin-PEG_{600}. While specific labeling of expressing cells (+) is achieved, no plasma membrane clustering of nanoparticle is observed if one of the two AuNPs lacks biotin-PEG_{600}. Blue arrowheads point towards individual AuNPs presented in the insets. Scale bars: left panels: 2 μm, right panels: 1 μm, insets: 100 nm.

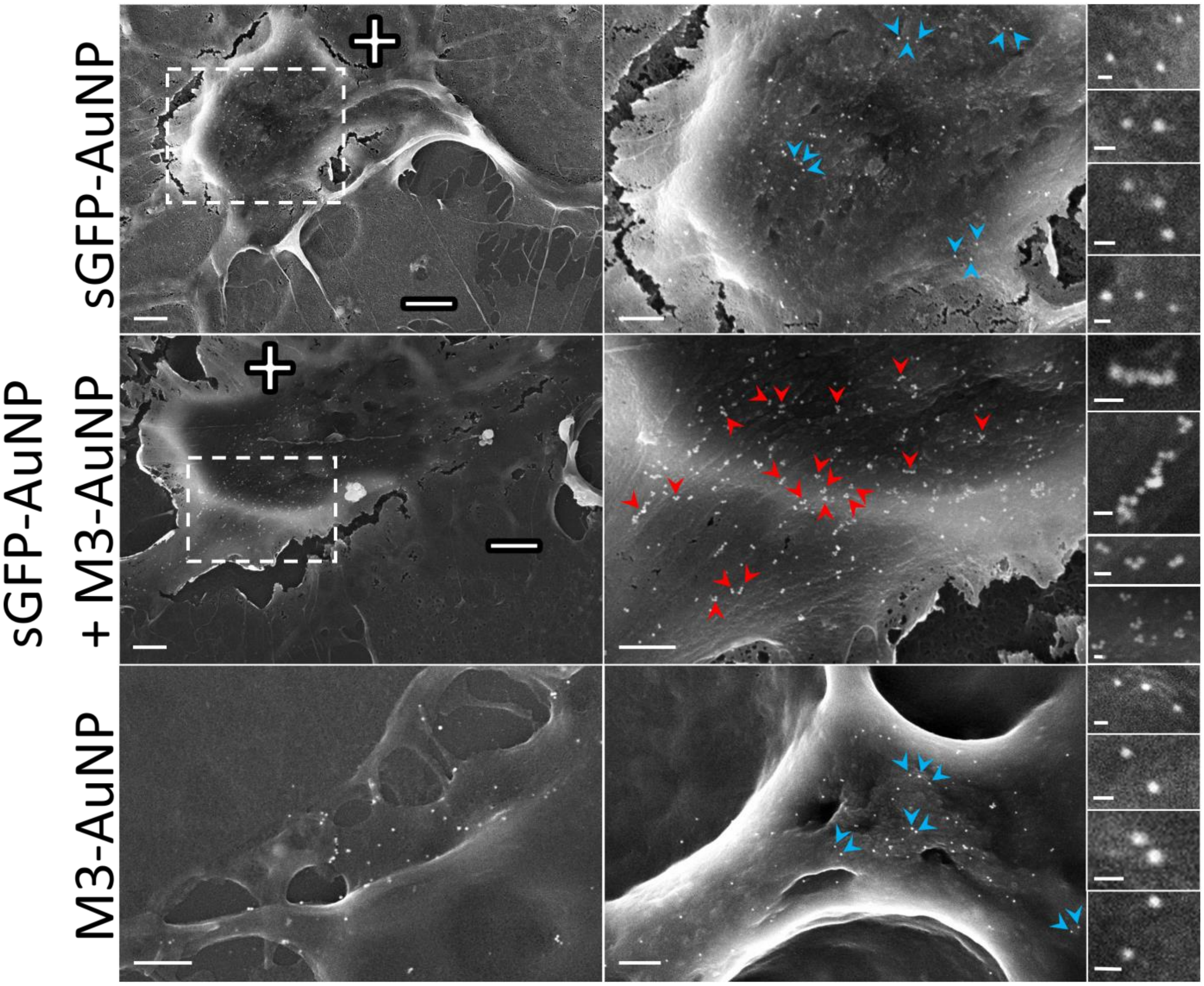

Supplementary Figure 23. SEM images of nanoclusters formed by biotin-PEG$_{2000}$-AuNPs on U2OS cells expressing both avidin fusions. Cells were incubated with sGFP-AuNP-biotin-PEG$_{2000}$ and M3-AuNP-biotin-PEG$_{2000}$ independently or together. As with AuNPs modified with biotin-PEG$_{600}$, efficient clustering is observed at the cell plasma membrane of expressing cells (+) for co-targeted nanoparticles only. Blue and red arrowheads point, respectively, towards individual or clustered AuNPs presented in the insets. Scale bars: Left panels: 2 μm, right panels:1 μm, insets: 100 nm.

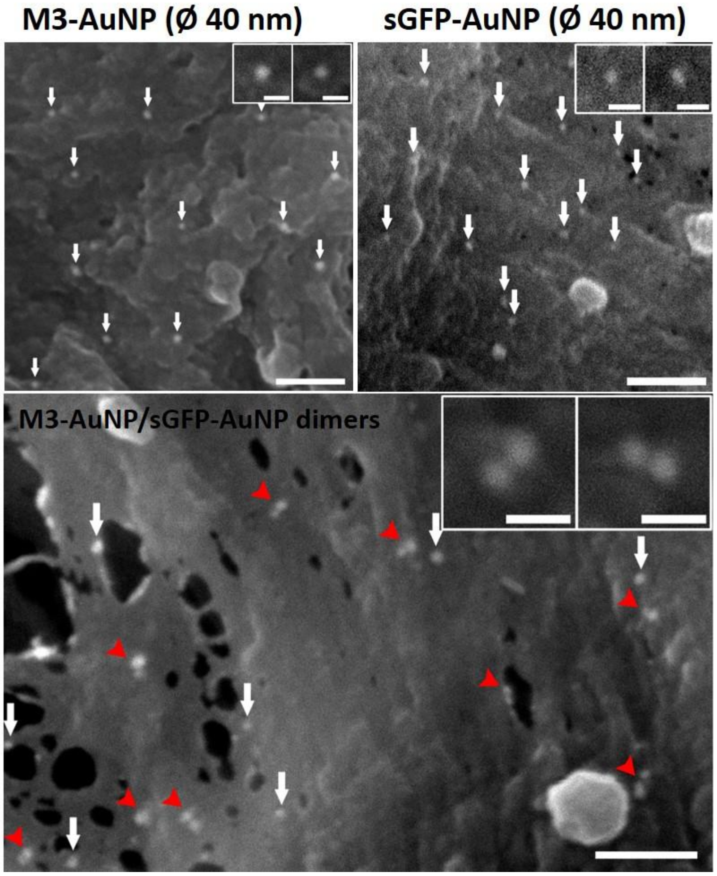

Supplementary Figure 24. SEM images of nanoclusters formed by biotin-PEG2000-AuNPs on HeLa cells expressing only the GPI-avidin fusion. Cells were incubated with sGFP-AuNP-biotin-PEG_{2000} and M3-AuNP-biotin-PEG_{2000} independently (top) or together (bottom). Clusters are primary dimeric (red arrowheads and insets) and residual monomeric AuNPs are observed at the plasma membrane (white arrows). Scale bars: 1μm; insets: 100 nm.

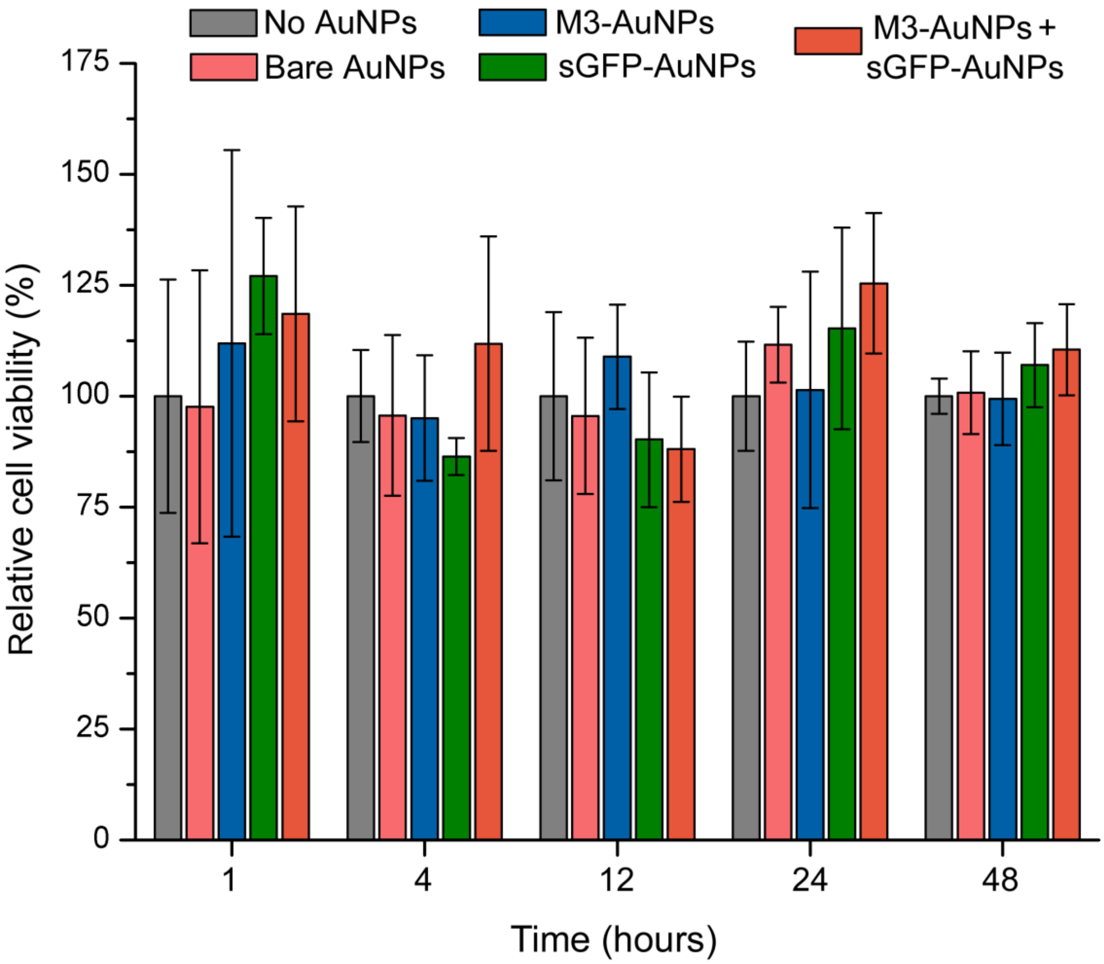

Supplementary Figure 25. MTT cell cytotoxicity assays for different AuNP formulations on U2OS cells co-expressing avidin fusions. Assays were performed at 1, 4, 12, 24 and 48 hours after incubation with AuNPs. Consistent with the inert and non-toxic nature of AuNPs, none of the AuNPs formulations induced cell toxicity compared to non-treated cells (No AuNPs). Assays were performed in four replicates per condition. Data represent the mean relative percentage of cell viability compared to cells without AuNPs ± standard error of the mean.

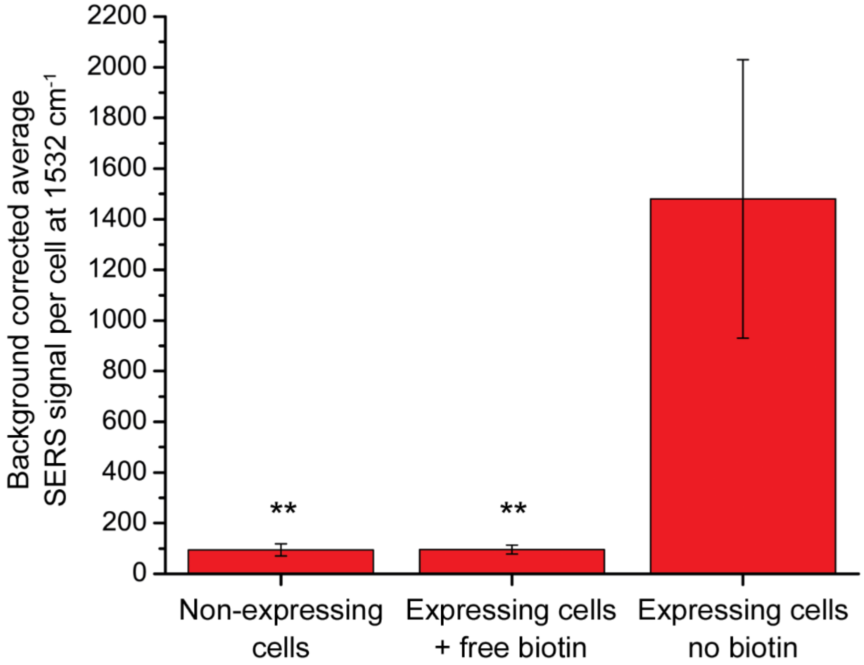

Supplementary Figure 26. Comparison of GFP SERS signal at 1527 cm^{-1} on targeted and non-targeted cells. GFP SERS signals were quantified on cells after co-incubation of biotin-M3-AuNPs + biotin-sGFP-AuNPs on U2OS cells not expressing avidin fusions (n=10), cells expressing avidin fusions and blocked with 25 µM free biotin (n=9) and cells expressing the avidin fusion without biotin blocking (n=11). Error bars represent the standard deviation of the mean. **: p<0.01, T-test.

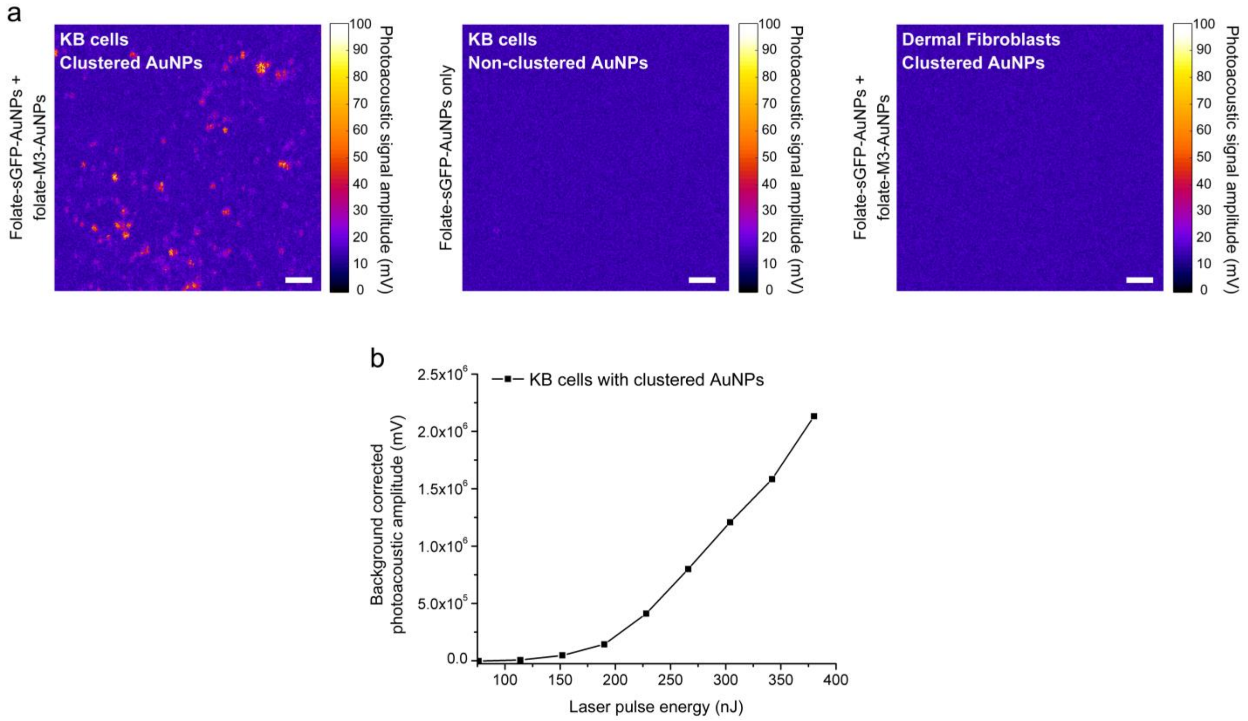

Supplementary Figure 27. Photoacoustic microscopy of folate-functionalized AuNPs targeted to carcinoma KB cells or primary dermal fibroblasts. a) Photoacoustic images acquire with a 532 nm pulse laser excitation at 190 nJ for KB cells targeted with both folate-sGFP-AuNPs and folate-M3-AuNPs (clustered AuNPs, left), for KB cells targeted with folate-sGFP-AuNPs only (non-clustered AuNPs, middle) and for dermal fibroblasts targeted with both folate-sGFP-AuNPs and folate-M3-AuNPs (clustered AuNPs, right). Scale bars: 50 µm. b) Photoacoustic signal amplitude from KB cells targeted with both folate-sGFP-AuNPs and folate-M3-AuNPs (clustered AuNPs) at increasing laser excitation energy. A non-linear photoacoustic response as a function of increasing excitation intensities is observed, consistent with the endocytosis of the assembled AuNP clusters, as generally observed for NPs targeted to folate receptors on cells.

Supplementary Table 1. AuNP cluster size distributions on targeted U2OS cells.

	sGFP-AuNP-biotin-PEG + M3-AuNP-biotin-PEG	sGFP-AuNP-biotin-PEG	M3-AuNP-biotin-PEG	sGFP-AuNP-biotin-PEG + M3-AuNP-PEG	sGFP-AuNP-PEG + M3-AuNP-biotin-PEG
**# of cells counted**	5	4	3	6	7
**# of AuNP/cluster**	**# of clusters (%)**				
Monomer	60.88	86.68	90.61	90.31	88.23
Dimer	26.97	10.56	7.96	8.16	9.54
Trimer	8.68	1.38	1.07	1.53	2.23
Tetramer	2.52	1.04	0.27	--	--
Pentamer	0.95	0.34	0.09	--	--

Note: U2OS cells co-express both the GPI- and transmembrane avidin fusions. "--": Not detected.

Supplementary References

1. He X, Bell AF, Tonge PJ. Isotopic Labeling and Normal-Mode Analysis of a Model Green Fluorescent Protein Chromophore. *The Journal of Physical Chemistry B* 2002, **106**(23)**:** 6056-6066.

2. Bell AF, He X, Wachter RM, Tonge PJ. Probing the ground state structure of the green fluorescent protein chromophore using Raman spectroscopy. *Biochemistry* 2000, **39**(15)**:** 4423-4431.

3. Lagant P, Vergoten G, Fleury G, Loucheux-Lefebvre MH. Raman spectroscopy and normal vibrations of peptides. Characteristic normal modes of a type-II beta turn. *Eur J Biochem* 1984, **139**(1)**:** 137-148.

4. Rygula A, Majzner K, Marzec KM, Kaczor A, Pilarczyk M, Baranska M. Raman spectroscopy of proteins: a review. *Journal of Raman Spectroscopy* 2013, **44**(8)**:** 1061-1076.

5. Wang H, Schultz ZD. The chemical origin of enhanced signals from tip-enhanced Raman detection of functionalized nanoparticles. *Analyst* 2013, **138**(11)**:** 3150-3157.

6. Stiufiuc R, Iacovita C, Nicoara R, Stiufiuc G, Florea A, Achim M, *et al.* One-Step Synthesis of PEGylated Gold Nanoparticles with Tunable Surface Charge. *Journal of Nanomaterials* 2013.

7. Barrow SJ, Funston AM, Gomez DE, Davis TJ, Mulvaney P. Surface Plasmon Resonances in Strongly Coupled Gold Nanosphere Chains from Monomer to Hexamer. *Nano Lett* 2011, **11**(10)**:** 4180-4187.

8. Chung T, Koker T, Pinaud F. Split-GFP: SERS Enhancers in Plasmonic Nanocluster Probes. *Small* 2016, **12**(42)**:** 5891-5901.
