# Supplementary material for: Cellular imaging by targeted assembly of hot-spot SERS and photoacoustic nanoprobes using split-fluorescent protein scaffolds
Source: Nat Commun. 2018 Feb 9;9:607. doi: 10.1038/s41467-018-03046-w (PMC5807522; doi:10.1038/s41467-018-03046-w)
Supplement: Supplementary file 2 — Descriptions of Additional Supplementary Files [file 41467_2018_3046_MOESM2_ESM.pdf]

Descriptions of Additional Supplementary Files

File Name: Supplementary Movie 1

Description: Total internal reflection fluorescence imaging of biotinylated M3-AuNPs targeted to HeLa cells expressing GPI-avidin fusion proteins. Biotinylated AuNPs are bound specifically to avidin expressing cells and individual AuNPs diffuse in the plane of plasma membrane. Acquisition: 100 ms/frame. Video playback: 33 frames/second.
